# Estimating energy consumption and GHG emissions in the U.S. food supply chain for net-zero

**DOI:** 10.1038/s41538-024-00346-y

**Published:** 2025-02-06

**Authors:** Kristina Armstrong, Wenquan Dong, Mingzhou Jin, Sachin Nimbalkar, Joe Cresko

**Affiliations:** 1 Manufacturing Energy Efficiency Research & Analysis, Oak Ridge National Laboratory, Oak Ridge, TN 37830 USA; 2 Ingram School of Engineering, Texas State University, San Marcos, TX 78666 USA; 3 Department of Industrial and Systems Engineering, The Institute for a Secure and Sustainable Environment, The University of Tennessee, Knoxville, TN 37996 USA; 4 Industrial Efficiency and Decarbonization Office, U.S. Department of Energy, Washington, DC 20585 USA

**Keywords:** Environmental impact, Agriculture, Economics

## Abstract

This work provides a database of the U.S. food system’s energy consumption and GHG emissions at the national and state levels by food supply chain (FSC) stage, fuel type, and food commodity. We estimate that the U.S. FSC consumed a total 4660 TBTU (4900 PJ) of site energy, 7130 TBTU (7500 PJ) of primary energy, and generated 970 MMT of GHG emissions in 2016. Among all the stages, on-farm production is the largest energy consumer (31% primary energy) and GHG emissions contributor (70%), largely due to raising animals. Optimizing distribution can reduce the stage’s energy consumption and GHG emissions and increase products’ shelf-life. Reducing food loss and waste is another good option, as it decreases the amount of food necessary to grow, thus impacting the overall FSC. The database can help stakeholders identify stage- and region-specific strategies and measures to curtail the environmental footprint of the U.S. food system.

## Introduction

In alignment with the goal of the Paris Agreement, which aims to limit global warming below 1.5 or 2 °C, the United States is committed to achieving a 50-52% reduction in economy-wide net greenhouse gas (GHG) emissions from 2005 levels by 2030, and ultimately reaching net-zero emissions by 2050^1^. Most efforts have focused on developing and promoting cross-sector solutions, such as clean energy technologies, though other innovations and behavioral changes are also being explored^2,3^. Even though the U.S. Food Supply Chain (FSC) accounts for almost 12% of the country’s annual fossil fuel budget while contributing over 18% of its annual GHG emissions^4^, less discussion is explicitly directed toward actions for the FSC. The FSC is widespread and has unique challenges that are not common across other industrial and commercial subsectors (e.g., direct crop and animal emissions, large waste streams, and low potential for electrification). However, some FSC-specific actions are being investigated and implemented. For example, the United States Department of Agriculture (USDA) launched a voluntary land conservation program in 2021 to remove environmentally sensitive land from agricultural production and plant species (e.g., trees) to improve water quality, increase soil carbon sequestration, and prevent soil erosion^5^. The United States Environmental Protection Agency (EPA) has also initiated various programs to promote more sustainable agricultural production (e.g., implementing biogas recovery technologies on farms to capture methane from livestock, reducing the application of chemicals in agriculture) to reduce GHG emissions, and increasing the use of renewable energy^6^. Moreover, the USDA and EPA have jointly announced the U.S. Food Loss and Waste (FLW) reduction goal^7^, which aims to halve FLW at the consumption stage to lower GHG emissions from organic waste management. The United States also collaborates with the United Arab Emirates on the Agriculture Innovation Mission for Climate, a global initiative to significantly increase public and private investment in climate-smart agriculture innovation for sustainable and resilient agricultural production^8^. However, most actions focus on reducing non-energy consumption-related emissions from on-farm production and consumer FLW management; solutions toward other FSC stages or across stages are still missing.

The U.S. administration is committed to building a sustainable, resilient, and inclusive domestic food system. This effort is essential to meet food security needs and provide healthy diets for all Americans while also addressing climate change. Reducing energy consumption and GHG emissions within the FSC is crucial to support this initiative and ensure the continued growth and prosperity of the country^9^. Quantifying the energy consumption and GHG emissions along the U.S. FSC and identifying the high-impact areas are crucial initial steps for achieving a net-zero U.S. food sector. This allows the development of regional- and stakeholder-specific strategies and targets that can help ensure a just and equal transition to a net-zero economy^10^.

As the literature review in Supplementary Note 1 shows, none of the existing studies provide a comprehensive and detailed accounting of the energy consumption and GHG emissions of the entire U.S. FSC (i.e., including both cradle-to-grave analysis and by food commodity groups and fuel types). Moreover, these studies all focused on the national level, with no state or regional analysis.

**Fig. 1:**
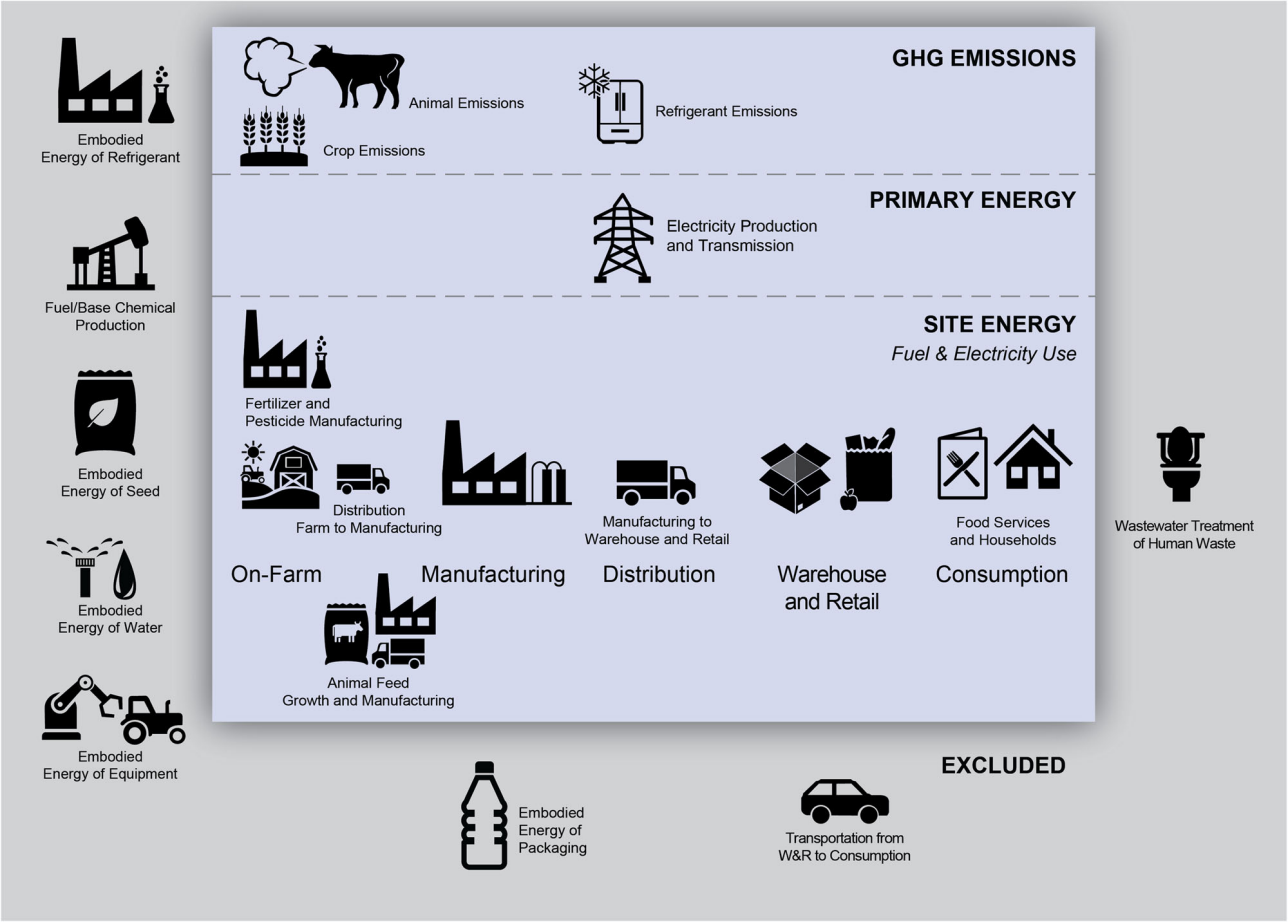
The boundaries of this analysis include the energy used and GHG emissions of the U.S. FSC but exclude embodied energies of chemicals, water, equipment, packaging, as well as other peripherals of the FSC.

This study is an extension of Dong et al.^11^ and considers the same five reduced commodity groups: grain and oil; fruits, vegetables, and nuts; dairy; sugar; and animal products (meat, and poultry, seafood, and eggs), matching available datasets (see Supplementary Note 3). The impacts of non-food agriculture and non-food uses of food commodities (e.g., oils and grains used for industrial uses or alcohol) were excluded from this work. Impacts from food commodities grown for livestock feed were included, but food crops grown for non-livestock (i.e., pets, horses) were not. The U.S. FSC is comprised of five stages: on-farm production (including pre- and post-farm activities like agricultural chemical manufacturing and farm-to-manufacturing transportation), manufacturing, distribution, wholesale and retail (W&R), and consumption (i.e., food services and households). While slightly different from some other FSC analyses such as FAOSTAT^12^ and EDGAR^13^ (two expansive datasets providing GHG emissions from the agri-food systems by countries including the U.S., see Supplementary Note 11 for further comparisons), these stages match the authors’ previous work^11^ and were chosen to provide a supply chain point of view to the FSC. By adopting this supply chain management perspective, the study not only facilitates comparisons of the U.S. FSC with those in other sectors but also underscores the importance of efficient supply chain management in achieving a sustainable U.S. FSC. The energy consumption at each stage is quantified in terms of site energy (i.e., energy consumed at the site of use) and then converted into primary energy. For this study, primary energy is defined as the energy required to produce the energy used on-site (e.g., electricity) plus any losses incurred through transmission and distribution; it is not true embodied energy as it does not include the energy required to refine fossil fuels or produce fertilizer or pesticide precursors and other starting materials and feedstocks. Additionally, the energy to produce the FSC infrastructure (e.g., farm equipment, manufacturing facilities, transportation equipment, cooking appliances) is considered to be outside the boundaries of this study and is excluded. For the GHG emissions analysis, direct emissions from fuel combustion (both on-site and for electricity generation), as well as direct emissions from crops and animals (e.g., soil management, enteric fermentation, manure management, rice cultivation) and fugitive emissions from refrigeration are included in this study. This study only focuses on the U.S. domestic energy consumption and excludes GHG emissions and the activities that occur outside the United States (e.g., international transportation and production of imported food), though the energy consumption and emissions from food that will ultimately be exported is still included, despite the food not being consumed within the United States. In addition, the energy consumption and GHG emissions for managing the FLW generated in each state are explored in more detail. For the end-of-life analysis, this study considers the same FLW definition and FLW management pathways as Dong et al.^11^. The detailed system boundary for energy consumption and GHG emissions by FSC stages are illustrated in Fig. 1 and described in more detail in “Methods” section and Supplementary Notes2–9. The full code and data used to develop the analysis and figures are on GitHub^14^.

This study aims to fill the gaps outlined above and aid the first steps of an equal transition to a net-zero economy by providing a comprehensive dataset illustrating the energy consumption and GHG emissions by the FSC stage, contributor (e.g., fuel, electricity, fertilizer), and food commodity group at the national and state levels.

## Results

**Fig. 2:**
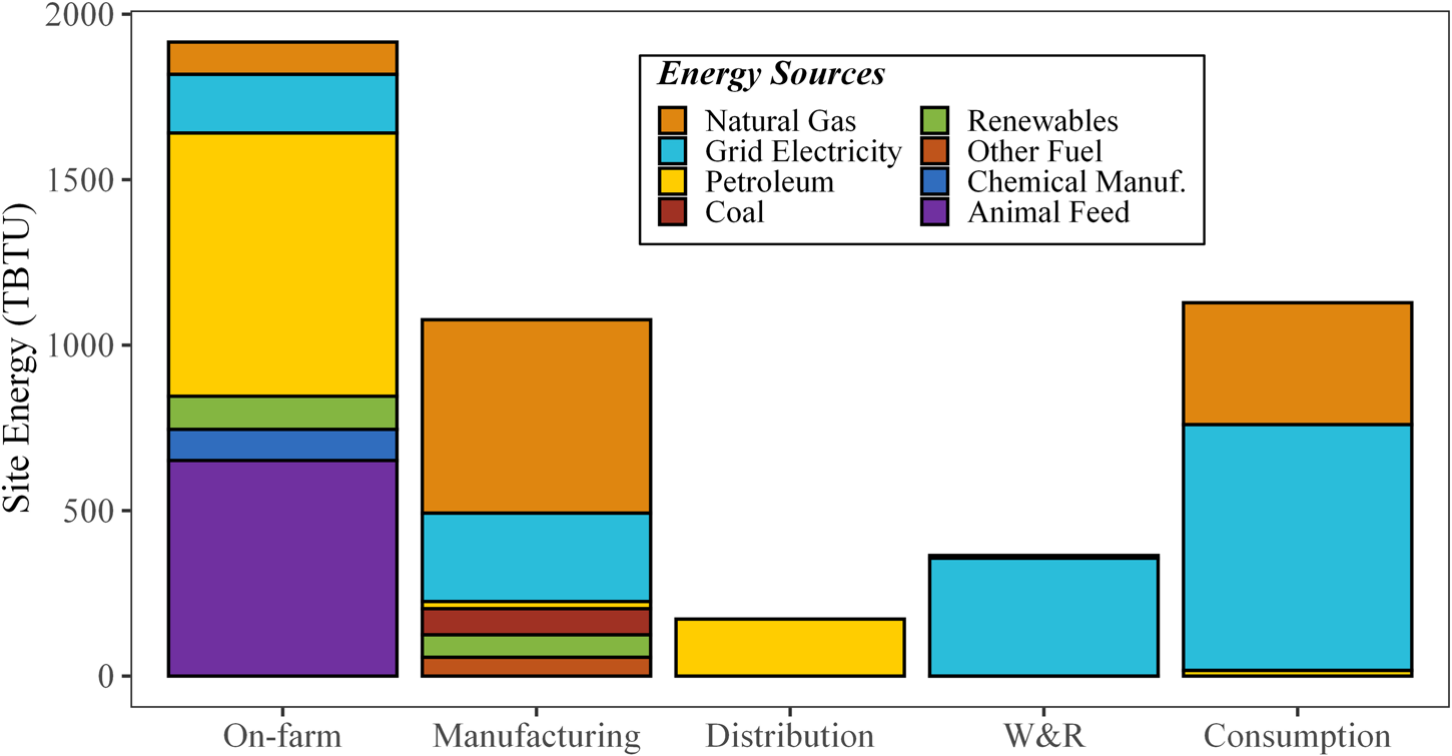
Site energy (TBTU) use of the 2016 U.S. FSC by stage and energy source.

**Fig. 3:**
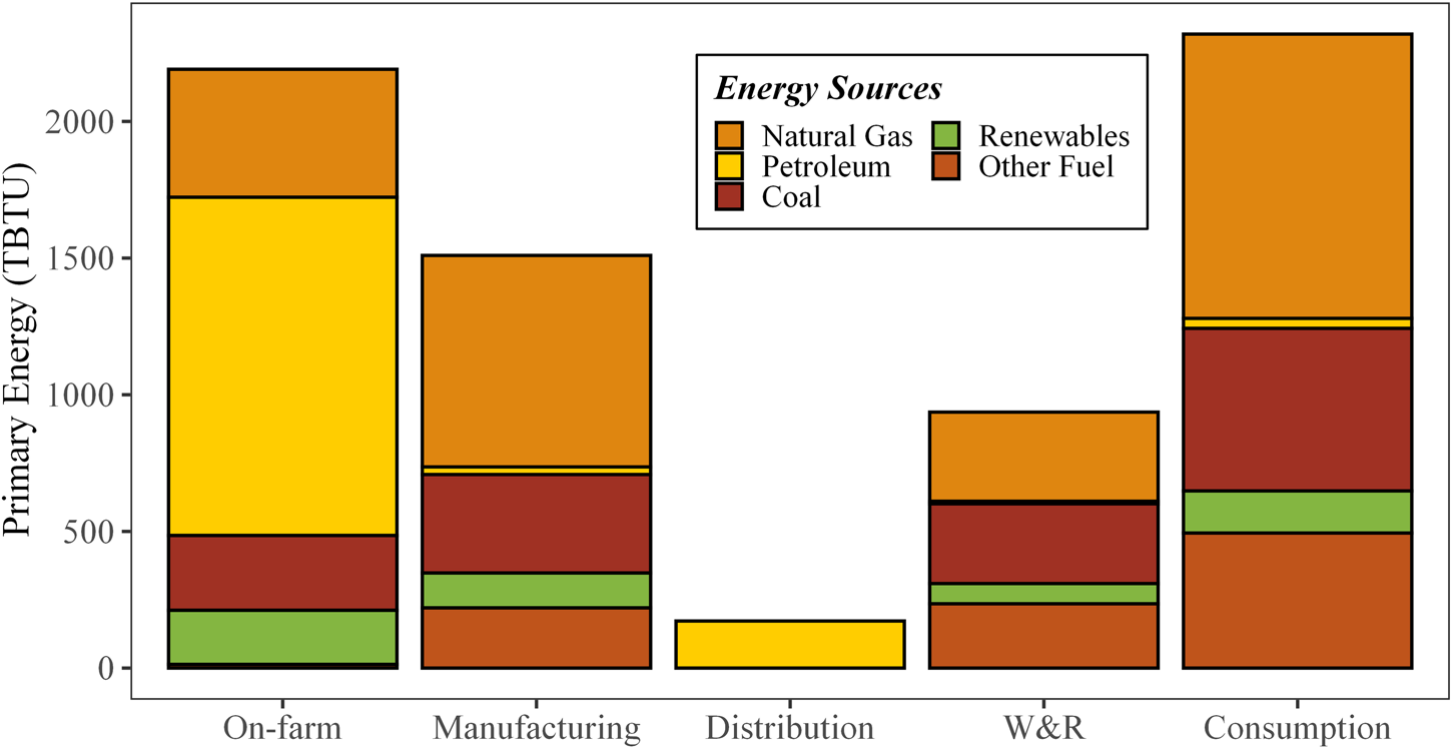
Primary energy (TBTU) of the 2016 U.S. FSC by stage and energy source.

**Fig. 4:**
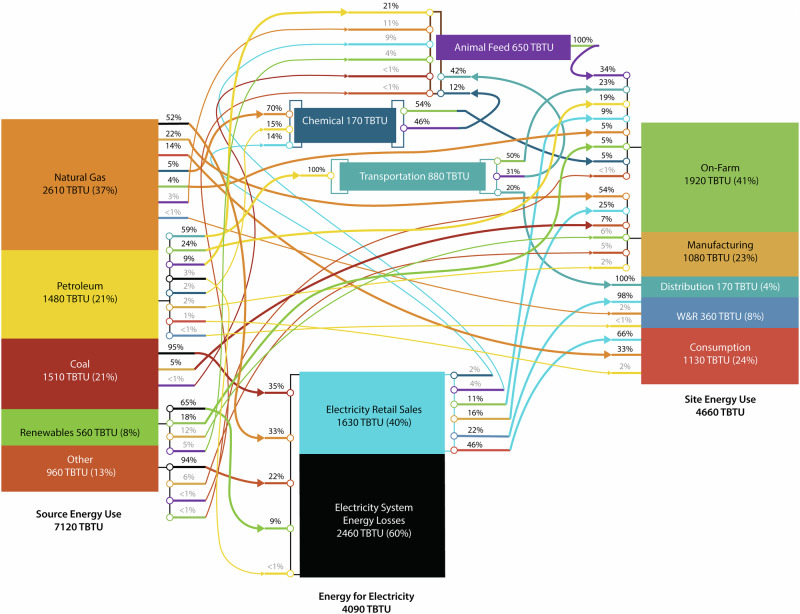
Overview of energy flows along the 2016 U.S. food supply chain, illustrating the energy flows into power generation, the transportation sector, animal feed manufacturing, and agricultural chemicals manufacturing.

**Fig. 5:**
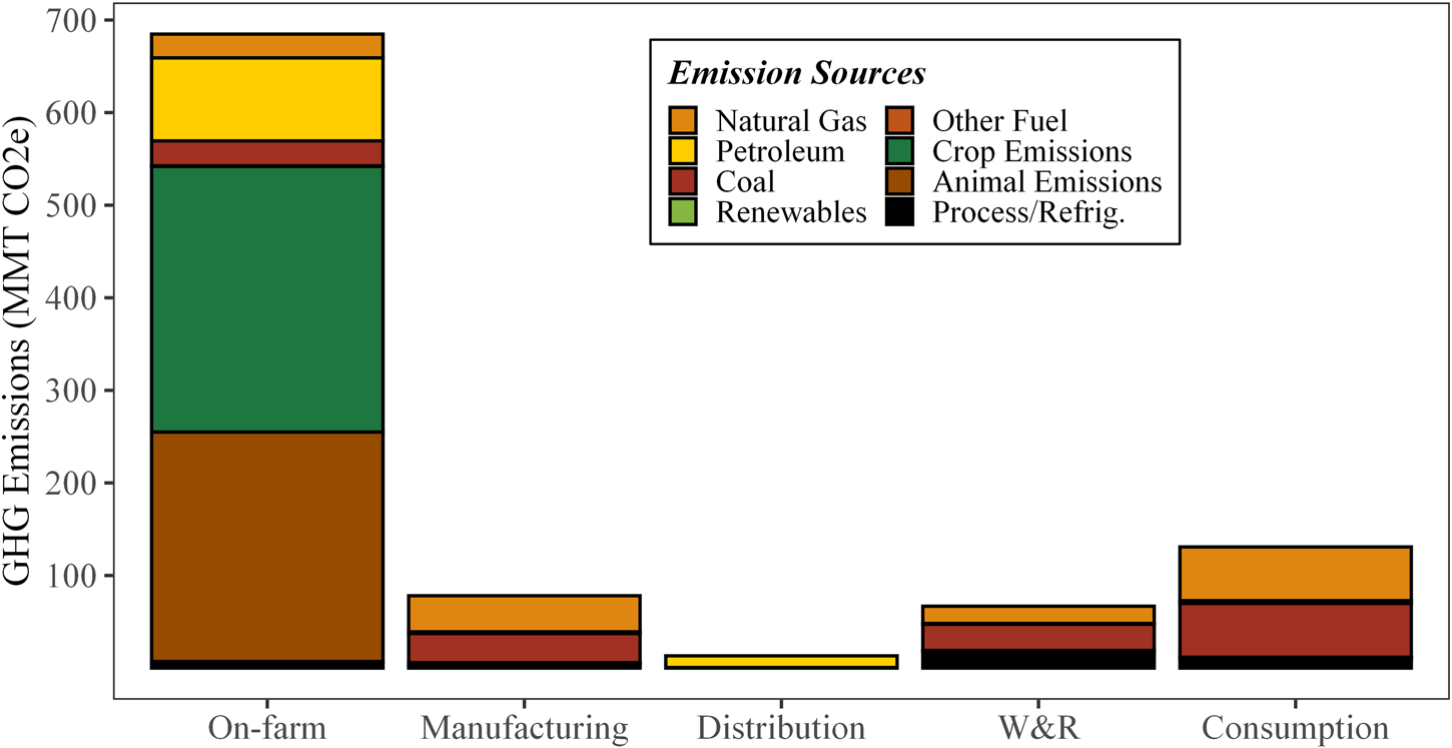
GHG Emissions (MMT CO_2e_) of the 2016 U.S. FSC broken down by stage and emission source.

According to the model created for this study, the entire U.S. FSC consumed 4660 TBTU (4910 PJ) of site energy, 7130 TBTU (7510 PJ) of primary energy, and originated 970 MMT CO_2e_ (based on 100-year global warming potentials using the Intergovernmental Panel on Climate Change [IPCC] Fourth Assessment Report [AR4] values^15^) of GHG emissions in 2016. This year was chosen for this modeling effort based on the large spread of date ranges of available data sources (2012–2020) and to align with Dong et al.^11^, see Supplementary Notes3–9. Figures 2–5 show the on-site and primary energy consumption and GHG emissions by each FSC stage and fuel type at the national level. When broken down by stages, site energy (Fig. 2) consumed at on-farm production ranked the highest (1920 TBTU, 2020 PJ, 41%) in the U.S. food system, followed by food consumption (i.e., food services and households) (1130 TBTU, 1190 PJ, 24%), food manufacturing (1080 TBTU, 1140 PJ, 23%), W&R (360 TBTU, 380 PJ, 7.8%), and food distribution (170 TBTU, 180 PJ, 3.7%). Electricity was the largest site energy source (33%) and was mainly used at the W&R and consumption stages (96% and 66%), followed by food manufacturing (25%) and on-farm production (9.2%). Natural gas (23%) and petroleum products (22%) were also major site energy sources for the U.S. food system. On-farm production and food distribution accounted for most of the petroleum combustion, and natural gas was mainly consumed in food manufacturing and food services. Agricultural chemicals manufacturing (fertilizer and pesticides) and animal feed production contributed to 2.0% and 14% of the site energy usage. Only the on-farm production and manufacturing stages had reportable volumes of on-site renewable energy use, representing a small portion (3.6%) of the energy used in the U.S. food system.

When converted to primary energy (Fig. 3), the difference in energy requirements between the on-farm production stage (2190 TBTU, 2310 PJ, 31%) and food consumption stage (2310 TBTU, 2320 PJ, 32%) shrinks dramatically. This change is caused by the high degree of electrification of the food consumption stage (66% of site energy supplied via electricity) and the low overall efficiency of the U.S. electricity generation industry (39.5% in 2016)^16^. The stages with the lowest degree of electrification (distribution, 0%, on-farm, 9%, and manufacturing, 25%), showed the least change between the site and primary energy breakdowns. The inclusion of electricity production in the energy breakdown for the FSC drastically increases the fractions of coal (1.7% to 20%), natural gas (23% to 33%), renewables (3.6% to 7.2%) and other energy sources (e.g., nuclear, 1.2% to 13%). This is also partially caused by the allocation of the primary energy that is used to create other site-level energy sources (e.g., animal feed, chemicals) to the fuels consumed. Figure 4 shows the way energy flows along the FSC, starting with the primary energy created from source fuels (left), various midpoint uses of energy (i.e., electricity generation, agricultural chemical manufacturing, and animal feed production), ending in the stage at which the energy is used (right). Here, the amount of energy needed to produce electricity is more obvious, with 60% (2461 TBTU) of the primary energy lost in generation, transmission, or distribution. When including this in the energy for each stage, fraction of energy use attributable to electricity increases for each stage. For instance, electrical-based energy increases to nearly half of manufacturing’s energy requirements (46%) and to 83% for the consumption stage.

Only 33% of the primary energy used towards the on-farm production of human food (i.e., electricity, fuel, on-site renewables) is used on-site, and the rest is consumed off-site for electricity generation, chemical manufacturing, animal feed manufacturing, or transportation of product to the manufacturing stage. For the remaining stages, the ratio of on-site to off-site energy consumption is directly related to the volume of electricity used. For example, in the consumption stage, 66% of the on-site energy used is electricity, therefore nearly half (49%) of the primary energy for the stage is consumed off-site.

**Fig. 6:**
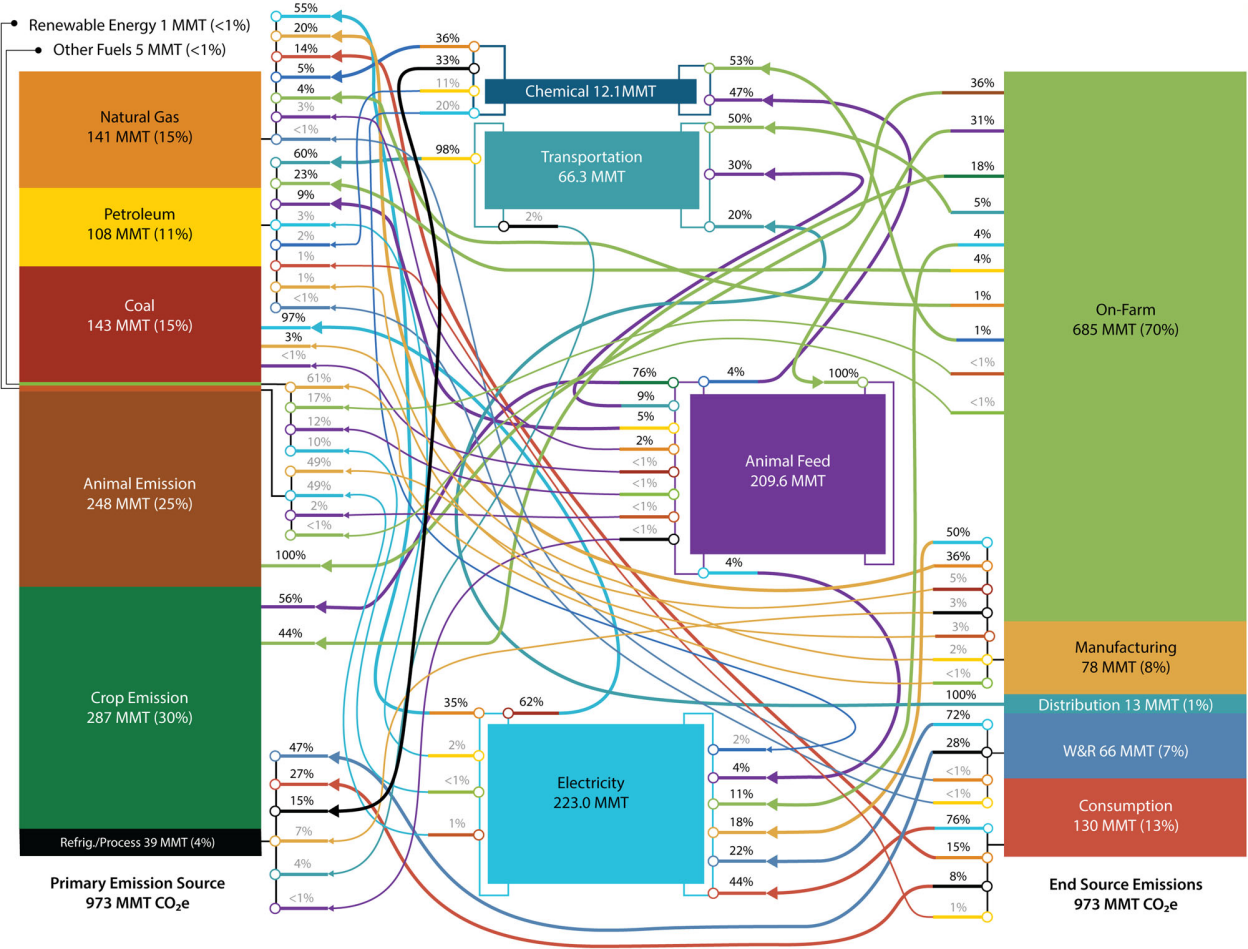
Overview of GHG flows along the 2016 U.S. food supply chain, illustrating the GHG emissions from power generation, the transportation sector, animal feed manufacturing, and agricultural chemicals manufacturing.

As shown in Figs. 5 and 6, on-farm production stage was the largest source of GHG emissions (679 MMT CO_2e_, 70%), followed by consumption (130 MMT CO_2e_, 13%), manufacturing (78 MMT CO_2e_, 8.1%), W&R (66.4 MMT CO_2e_, 6.9%), and distribution (13.2 MMT CO_2e_, 1.4%). Most of the GHG emissions at the manufacturing, distribution, W&R, and consumptions stages are due to primary energy consumption with a small portion (3.3–28%) due to refrigerant fugitive emissions. Unlike the other stages, most of the on-farm emissions are caused by crop- and animal-based emissions (mostly methane and nitrous oxide; 18% from human food crops, 24% from animal feed crops, and 36% directly from animals) rather than energy consumption. Overall, energy-related GHG emissions is dominated by coal and natural gas, again, due to their high use in electricity production. Finally, refrigerant fugitive emissions account for 3.4% of the total FSC GHG emissions; while this is relatively small, it does constitute 28% and 8% of the W&R and consumption stages’ emissions. The high W&R and consumption fugitive emissions is likely caused by the use of relatively less efficiency cooling systems in retail and food services (15–35% operating losses per year) compared to industrial refrigeration systems (used in manufacturing and cold storage warehouse, 15–25% operating losses per year)^17^. Like Fig. 4, Fig. 6 highlights the intermediate sources of emissions (i.e., electricity, chemical manufacturing, animal feed production, transportation), as well as the high-level emission sources for the entire FSC.

The following subsections provide more detailed results for each FSC stage, the energy consumption and GHG emissions associated with FLW disposal, and the geographic distribution of energy consumption and GHG emissions at the state level.

### GHG emissions and energy consumption breakdown by commodity—on-farm production

This study defines on-farm production as the agricultural activities for producing crops, farm animals, and seafood for human consumption at the farm level and those occurring before reaching food manufacturers’ gate (including transportation of food to manufacturers, the production of fertilizer, and the growth and production of animal feed). As defined by the U.S. Energy Information Administration (EIA)^18^, the on-farm energy consumption includes both direct and indirect energy consumption. On-farm direct site energy includes the use of natural gas, petroleum, grid electricity, and on-site generated renewable energy for on-farm agriculture activities. Indirect site energy includes the energy required to manufacture fertilizers and pesticides, the shipment of agricultural materials to manufacturing sites, as well as the agricultural production, transportation, and manufacturing of animal feed. This study also allocates the energy for chemical and animal feed production to their source fuels.

On-farm production was the largest contributor to site and primary energy usage and GHG emissions among all five FSC stages in 2016. This analysis estimated on-farm production used 1920 TBTU of site energy (2190 TBTU primary energy) and emitted 680 MMT CO_2e_. Overall, the primary energy consumption breakdown at this stage was very similar to site energy consumption due to the low electrification rate. This analysis also details the energy use and GHG emissions for the different commodity groups.

**Fig. 7:**
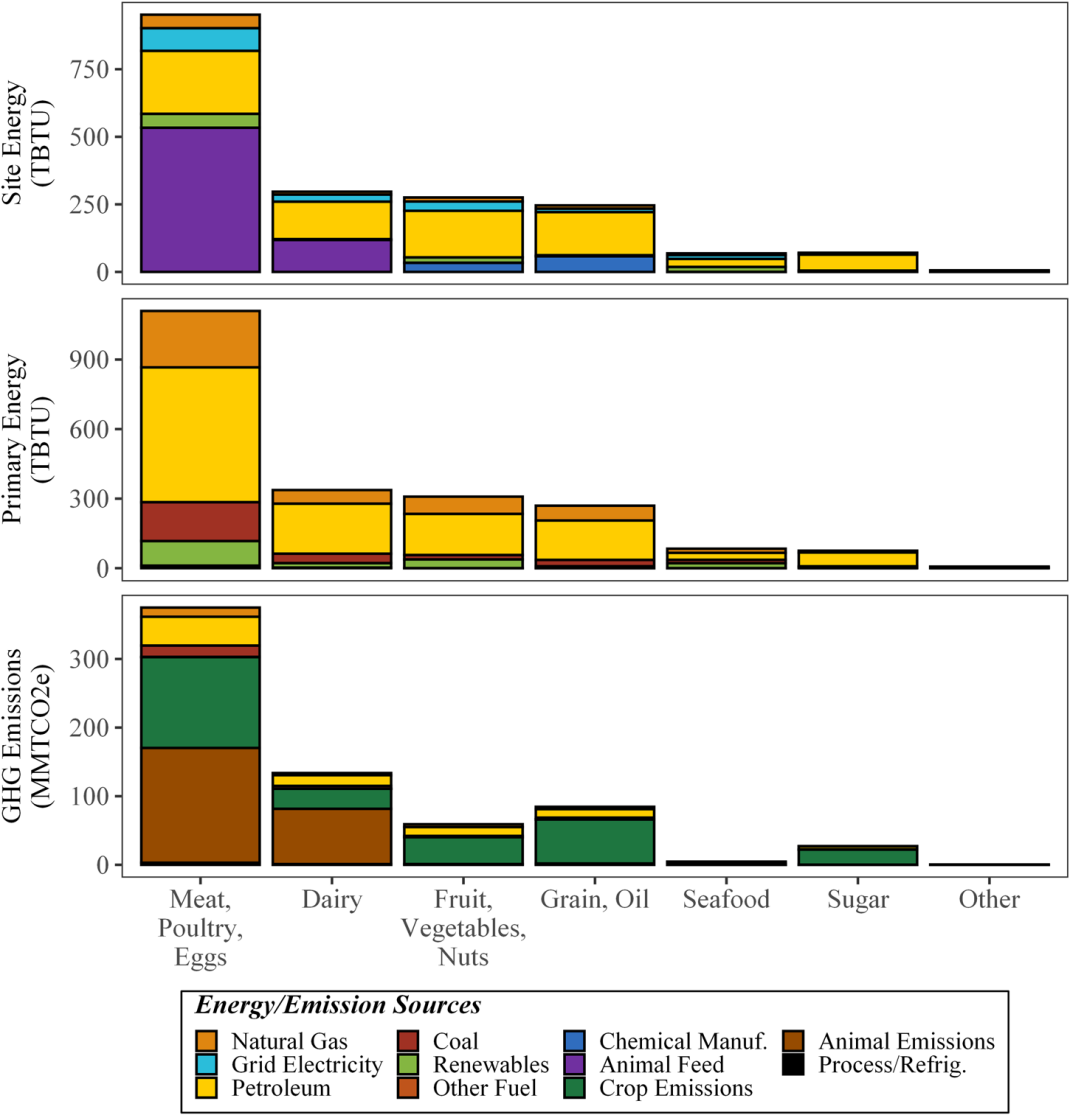
Site and primary energy use (TBTU) and GHG emissions (MMT CO_2e_) of the on-farm stage in the 2016 U.S. FSC, broken down by energy or emission source and commodity group.

As demonstrated by Fig. 7, commodities that involve raising land animals (i.e., meat, poultry, eggs and dairy products) are the two largest energy users (50% and 16% of stage site energy and 51% and 15% of stage primary energy, respectively). Such high energy use is caused by the inclusion of the growth, manufacturing, storage, and transportation of animal feed (56% of site energy use for meat, poultry, and eggs and 40% of site energy use for dairy products). Growing fruits, vegetables, and nuts (14% of both site and primary) have the next highest energy demand, followed by grain and oil crops (12% of both site and primary). The other food commodities (i.e., seafood, sugar, and others) all together contribute only 7% of site and primary energy consumption, mainly due to their low demand.

Looking at how the energy is used on-site, petroleum combustion, which is used for farm equipment, heating, transportation from farm to manufacturing, was the highest energy use (42%), followed by energy consumed for animal feed farming and manufacturing (34%). Agricultural chemicals manufacturing (i.e., pesticides and fertilizers) is the third largest energy consumer (4.9%) of on-farm site energy consumption, which is mostly used for growing grain and oil crops, including animal feed. Grid electricity (9.2%), renewables (5.2%), natural gas (5.1%), and other fuels (less than 0.01%) make up the remainder.

Again, Fig. 7 shows that the on-farm GHG emissions were dominated by crop and animal emissions (42% for human and feed crops, e.g., rice cultivation, soil management and 36% for animal-based, e.g., manure management, enteric fermentation), which are non-energy-based emissions. On-site energy use (fuel combustion or electricity use) only accounted for 8.8% of total emissions. Crop emissions, for this study, are from a variety of sources related to the fertilization and cultivation of crops and cropland. Crop emissions do not include the manufacturing of fertilizers and pesticides (those are allocated under chemicals manufacturing for this study) but do include direct CO_2_ releases from urea (the conversion of urea, in the presence of water and urease enzymes, into ammonium, hydroxyl ions, and bicarbonate, which then evolves into CO_2_) and lime (crushed limestone and dolomite reacting with soil-based hydrogen ions to form CO_2_). Crop emissions also include methane emissions from rice cultivation (from methanogenic bacteria in flooded rice fields), nitrous oxide (N_2_O) created from the microbial processes of nitrification and denitrification of mineral nitrogen in the soil and released via various soil management activities, and methane and N_2_O releases from the burning of agricultural residues. Animal emissions are more straightforward, either methane releases from enteric fermentation (i.e., gases from digestion) or methane and N_2_O releases from manure management. While often used as a crop fertilizer, manure was allocated as an animal-based emission in this study. The largest GHG emission from these sources is N_2_O from soil management, contributing 330 MMT CO_2e_ (95% non-energy-based N_2_O, 55% non-energy-based emissions). Methane is the second largest GHG emission, with 245.3 MMT CO_2e_, 70% of which is from enteric fermentation (24% from manure management and 6% from rice cultivation). Details on how these emissions were allocated to different commodities and geographic regions can be found in Supplementary Note 3.

### GHG emissions and energy consumption breakdown by commodity—food manufacturing

Food manufacturing includes both food processing (e.g., cooking, cutting, trimming, slaughtering) and packaging (e.g., sorting, boxing, wrapping). However, the energy consumption and GHG emissions of producing food packaging materials were beyond the scope of this work. Energy use at this stage resembles that of other types of manufacturing, with substantial energy being used for process heating (directly or via steam), process cooling, motor-driven systems (e.g., pumps, fans, conveyors), and non-process energy (e.g., facility HVAC and lighting).

**Fig. 8:**
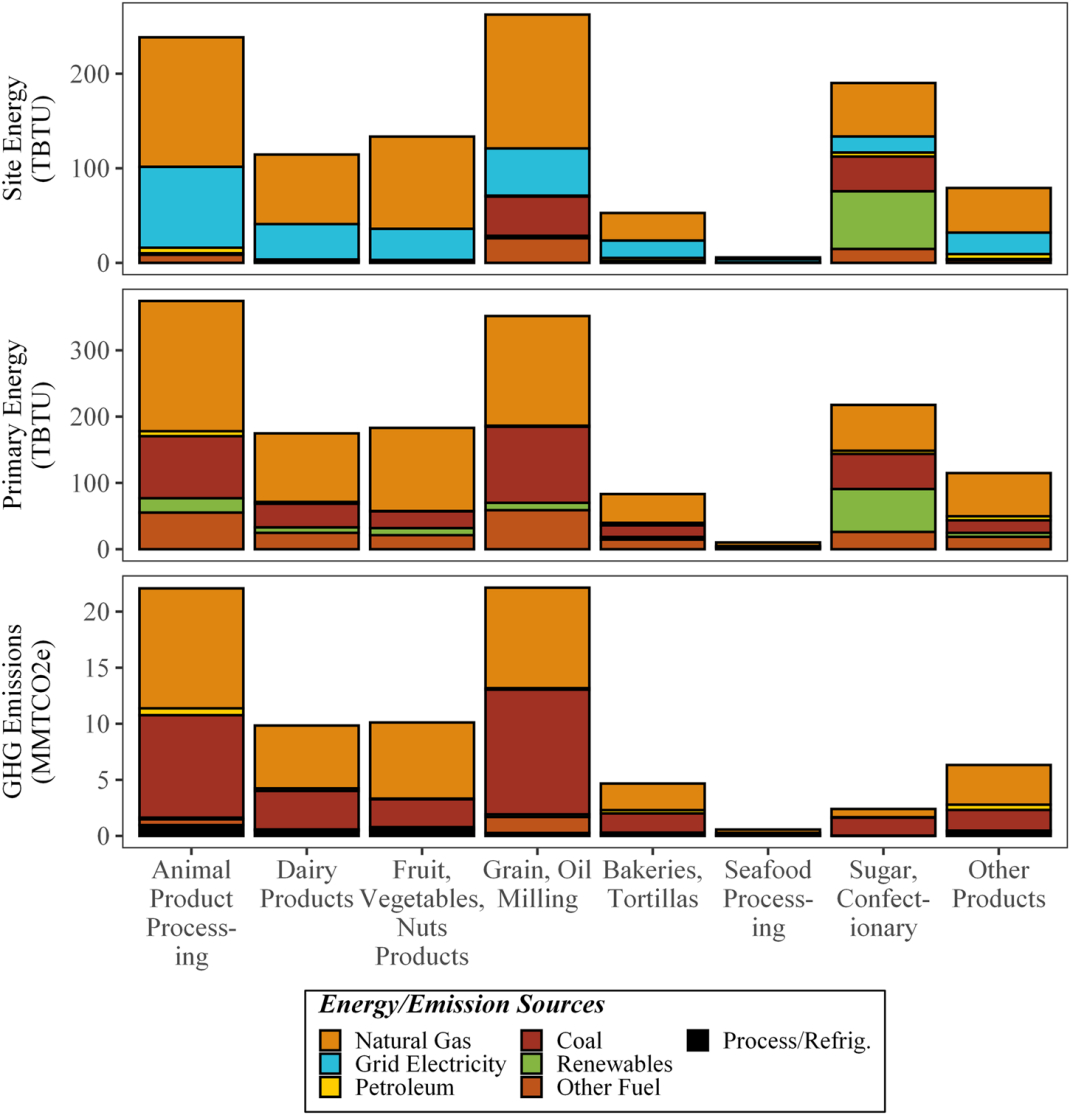
Site and primary energy use (TBTU) and GHG emissions (MMT CO_2e_) of the manufacturing stage of the 2016 U.S. FSC, broken down by energy or emission source and commodity group.

Figure 8 shows the estimated on-site (1080 TBTU) and primary (1510 TBTU) energy consumption and GHG emissions (78 MMT CO_2e_) at the manufacturing stage. This stage requires energy-intensive refrigeration and process heating, along with motor-driven manufacturing equipment (e.g., pumps, fans, movers). While the breakdown of energy users is not explored further in this analysis, the U.S. Manufacturing Energy Consumption Survey (MECS)^19^ and subsequent analyses show that these systems collectively are responsible for 60% of the energy used by the food and beverage manufacturing sector (37% for process heating, 10% for process cooling and refrigeration, 15% for motor-driven equipment) and non-process energy requires about 20%. Like the on-farm stage, manufacturing of animal-related products (i.e., animal products processing and dairy products processing) contributed substantially to the energy consumption (22% and 11% on-site energy, 25% and 12% primary energy, respectively) and GHG emissions (28% and 13%, respectively) of food manufacturing. Grain and oil milling, sugar and confectionery, and produce (e.g., fruit, vegetable, nuts) processing were also large contributors to energy use, though sugar and confectionery had substantially lower GHG emissions due to the use of waste bagasse for energy generation. Seafood processing contributed the least energy consumption and GHG emissions (both less than 2%) due to the low demand.

Representing 54% of the stage’s site-energy use, natural gas is the most used on-site fuel for all commodities, ranging between 30% and 73%, except for sugar and confectionary. Electricity, comprising 25% of the stage’s site-energy use, is usually the second most used energy source across all communities. Most commodity subsectors do not use coal, with the interesting exceptions of sugar and confectionary processing (19%) and grain and oil milling (16%). In terms of primary energy and GHG emissions, the highest impact comes from the consumption of natural gas and coal for electricity generation. These two fuels accounted for about 90% of the GHG emissions and about 75% of the primary energy at the food manufacturing stage.

### GHG emissions and energy consumption breakdown—distribution, W&R, and consumption

The distribution energy use and GHG emissions described in this subsection only covers the transportation of food products from manufacturers to W&R sites. Transportation from farms to manufacturers is part of the on-farm stage and transportation from W&R sites to consumption sites is not considered in this analysis. The food distribution stage was the smallest contributor to total FSC energy, contributing 170 TBTU to both site and primary energy consumption (3.6% and 2.4%, respectively) and 13.2 MMT CO_2e_ to GHG emissions (1.4%). This analysis assumes that petroleum is the only energy source (thus having an equal site and primary energy), as electric vehicles were not implanted into food transportation at of the time of the data’s collection. The calculation only includes intrastate transport of goods and excludes the energy contributions of imports and exports. Most of the energy is dedicated to transporting dairy products (25%), produce (48%), and animal products (21%), which also require extra power and produce fugitive emissions due to their temperature requirements. While the distribution stage is only a minimal contributor to the total FSC energy and GHG emissions, it serves as the connection between food manufacturers and W&R, and thus end consumers, and is the key time bottleneck. Shortening the food transportation distance, such as by decentralizing the FSC, can help increase food products’ shelf-life, therefore reducing the food loss and waste for the distribution, W&R, and consumption stages, as well as food demand at each FSC stage.

The Wholesale and Retail (W&R) stage includes the activities of food storage and sale before the consumption stage. Wholesalers facilitate the sales between manufacturers and retailers or food services, taking responsibility for the storage and shipment. Retail is the sale of food primarily to consumers that is consumed off-premises (e.g., grocery stores). At this stage, the energy consumption primarily stems from electricity use, while GHG emissions originate from fuels and fugitive emissions from cold storage. This analysis estimates that W&R stage consumed 360 TBTU (7.7%) of site energy and 940 TBTU (13%) of primary energy, with 66.8 MMT CO_2e_ of GHG emissions (6.8%) in 2016. Digging deeper, this study estimated the energy consumption and GHG emissions for warehouses and retail stores separately. Overall, food warehouses were estimated to use less than 1% of the U.S. FSC energy, whereas the retail substage uses 7% of site energy and 12% of primary energy. For this stage, electricity was the major site energy source used. For warehouses, cold storage warehouses mostly use electricity (77% site energy), with dry warehouses having a bit more equal split (with electricity comprising 56% of the energy use). Retail stores were assumed to use only electricity. In addition to GHG emissions from fuel combustion and electricity production, refrigerant emissions contributed 1.2 MMT CO_2e_ from warehouses (mostly cold storage) and 17 MMT CO_2e_ from retail, representing 28% of the total emissions from the W&R stage.

**Fig. 9:**
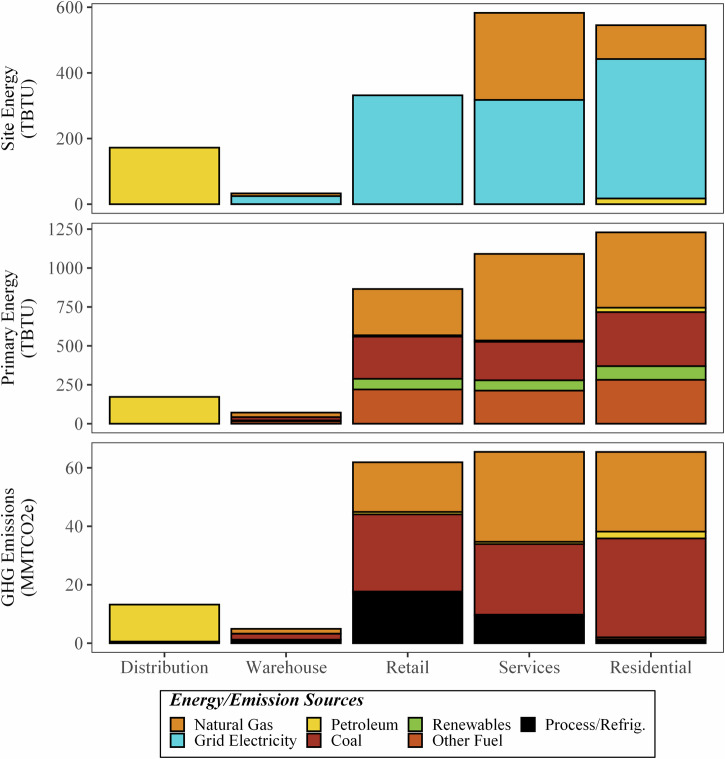
Site and primary energy use (TBTU) and GHG emissions (MMT CO_2e_) of the final three stages of the 2016 U.S. FSC—distribution, W&R, and consumption, broken down by energy or emission source and substage.

Finally, this study also estimated the energy consumption and GHG emissions at the consumption stage to be 1130 TBTU site, 2320 TBTU primary, 130 MMT CO_2e_, as well as exploring food services and residential impacts separately. Consumption designates where the food is consumed by people, including the food purchased and consumed in households or from food services (e.g., restaurants, hospitals, and government facilities). We consider food storage and preparation as the major energy users (i.e., electricity, natural gas, and propane) and GHG emissions causes at the consumption stage. As shown in Fig. 9, food services had a higher site energy consumption (580 TBTU) than the residential substage (550 TBTU). However, when considering the primary energy, the residential sector had a higher total energy consumption and similar GHG emissions (1230 TBTU compared to 1090 TBTU, both with about 65 MMT CO_2e_), due to the higher electrification of the residential subsector. Additionally, other studies^9^ have estimated that only 35% of food was consumed at food services, indicating a lower energy efficiency in food services than residential preparation. While not explored in this work, this lower efficiency in food services could be caused by the differences in energy sources (electricity or natural gas for cooking) or differences in refrigeration systems (e.g., larger, less full, walk-in freezer units compared to smaller home refrigeration units). Finally, compared with the residential subsector, food services had a more fugitive refrigerant emissions (9.2 MMT CO_2e_ compared to 1.3 MMT CO_2e_). Figure 9 illustrates the energy use and GHG emissions of these final three stages, broken down by fuel consumed and substage.

### GHG emissions and energy consumption of FLW disposal

As discussed in Dong et al.^11^, 30% of the food planted or raised was lost or wasted (i.e., FLW) in the U.S. in 2016. Some of that FLW is recovered/recycled (21%) as food donations, animal feed, feedstock for industrial process or anerobic digestion (AD), or composted. The rest ends up in landfills or wastewater treatment facilities (WWT; via in-sink disposal) or is incinerated (9.2%). Some of the energy required to dispose of the food is already included in this analysis. For example, food donation is already present in consumption, animal feed manufacturing includes that from human FLW, and offsets from fertilizer generation in AD systems are part of the fertilizer used. However, other disposal methods are not an integrated part of the FSC.

**Fig. 10:**
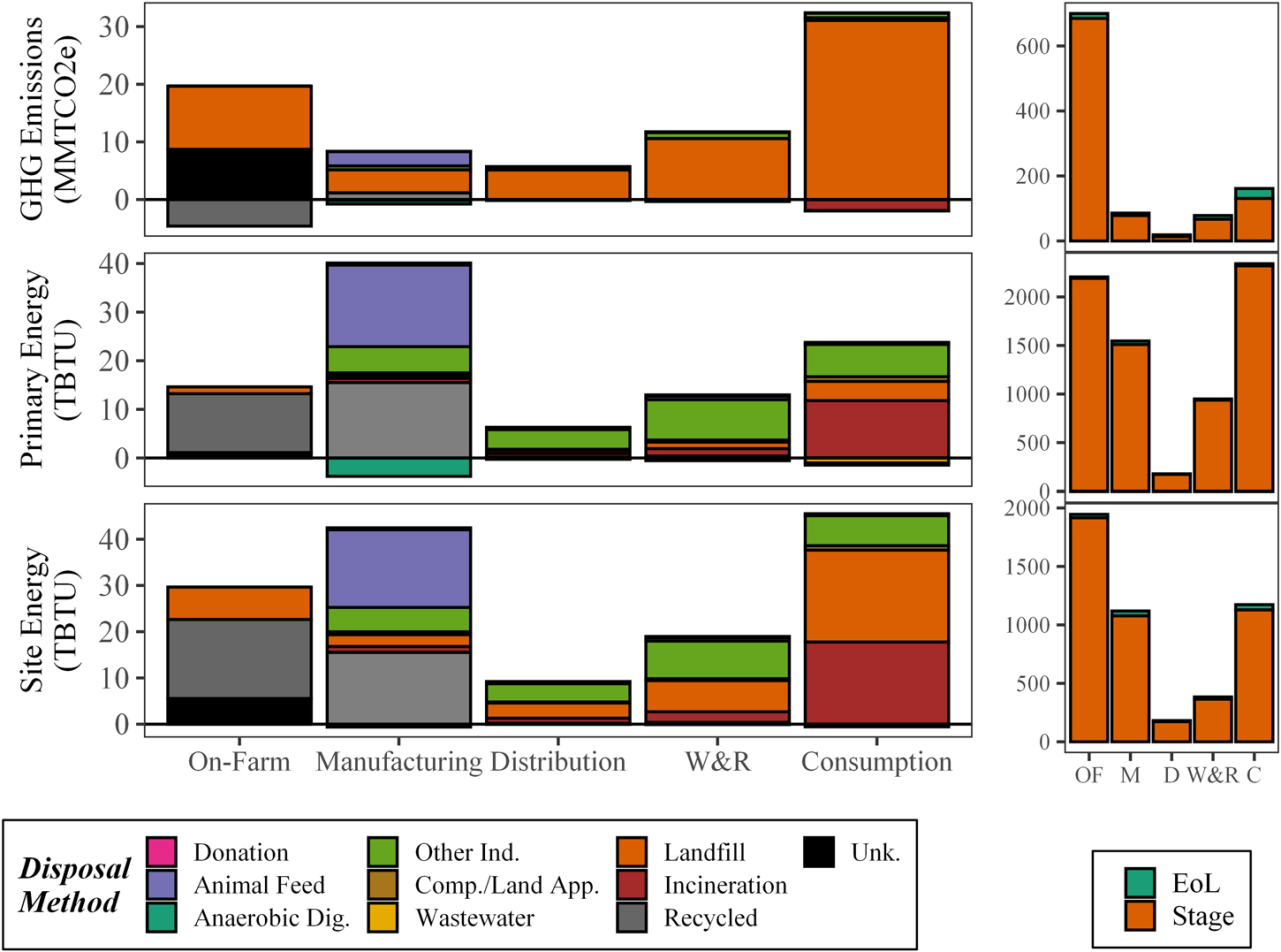
Net additional end-of-life site/primary energy and GHG emissions for each disposal pathway in the 2016 U.S. FSC, broken down by contributor and stage (left) and shown as part (green segment) of the total (orange segment) energy and emissions (right, OF: On-Farm, M: Manufacturing, D: Distribution, C: Consumption).

Figure 10 shows the net energy impacts of FLW disposal methods, expect for those already considered to be part of the FSC. Landfill, AD, WWT, and incineration are modeled as having an energetic offset, as they can produce more electricity than they consume, leading to all stages having a smaller primary energy than site energy. As mentioned above, while AD, composting, and WWT may also have fertilizer offsets, those are already integrated into the above analysis of the FSC. The energy for FLW disposal via food donation and animal feed have an energetic addition for the collection and transportation of FLW from the generation point to the utilization point (i.e., food banks followed by consumption and animal feed manufacturing, respectively).

Overall, the incineration and landfilling FLW from the consumption stage are two of the largest contributors to the energy consumption for FLW disposal, despite their electricity offsets. Using FLW as feedstock for bio-based industrial products is also among the top FLW management energy users for all stages, though bio-based products, which are beyond the scope of this analysis, would have offsets make this impact smaller. The energy to transport animal feed from FLW generation to animal feed manufacturing sites is another major contributor, due to the large amount of FLW managed via this method. The consumption stage has the highest contribution to FLW disposal energy use (44.9 TBTU site and 22.3 TBTU primary), though it is only a small portion of its energetic contribution to the FSC (3.8% site, 1.0% primary). Overall, the energy required for additional disposal processing is a small portion of the FSC energy (3.0% of site energy and 1.3% of primary energy) but needs to be considered for a complete analysis of the FSC. Finally, due to the high fugitive methane emissions of landfills, disposal can be a significant contribution to the GHG emissions of the overall FSC (almost 7% of the entire FSC and 19% and 30% for the consumption and distribution stages). Other disposal methods are substantially less impactful on the FSC GHG emissions, for example incineration actually provides a small net negative impact due to its offsets from generation low carbon electricity (due to the biogenic CO_2_ emissions being discounted).

### GHG emissions and energy consumption at the state level

**Fig. 11:**
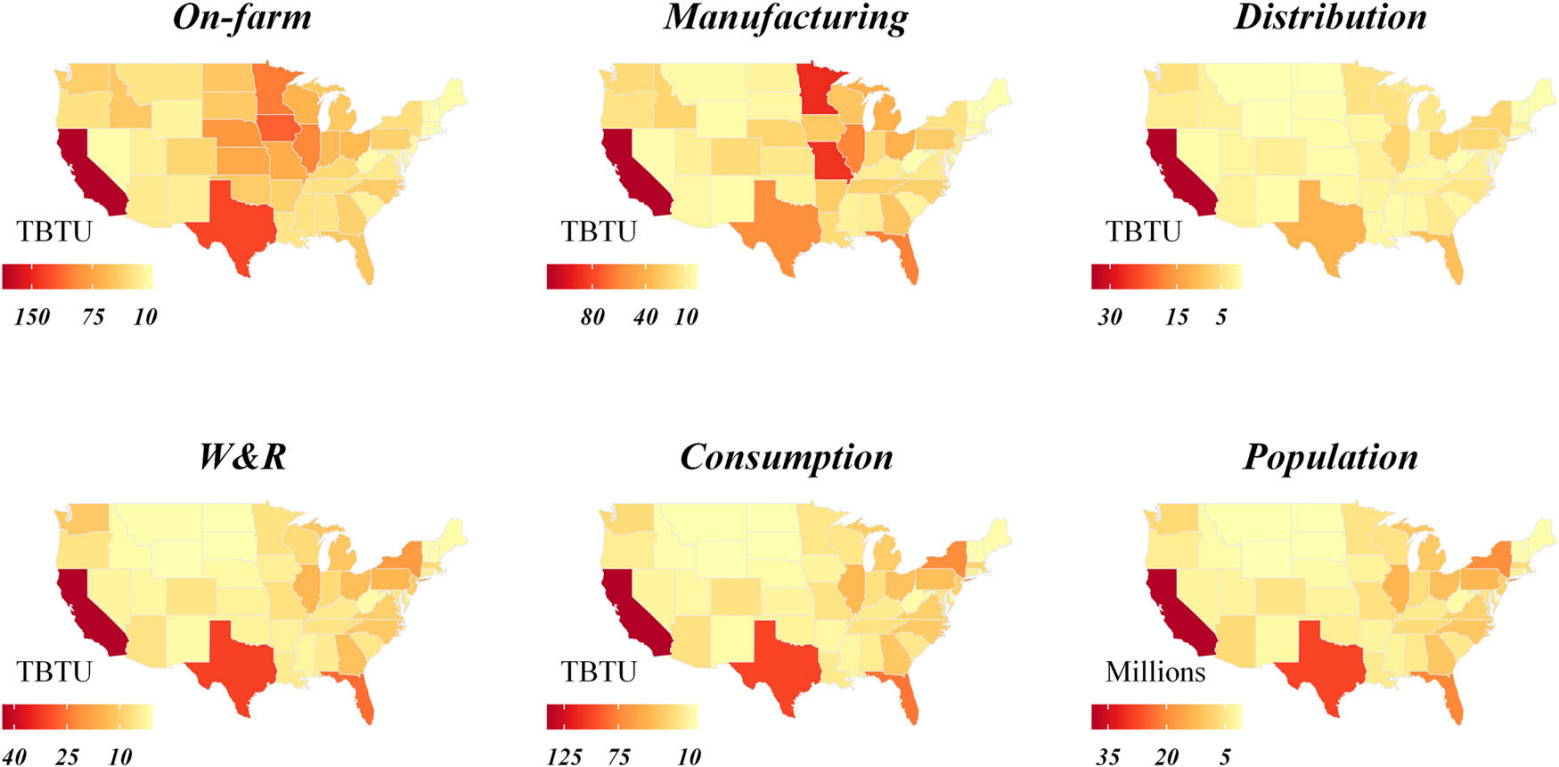
State-level on-site energy use for each stage of the 2016 U.S. FSC, plus 2016 U.S. population^16^.

**Fig. 12:**
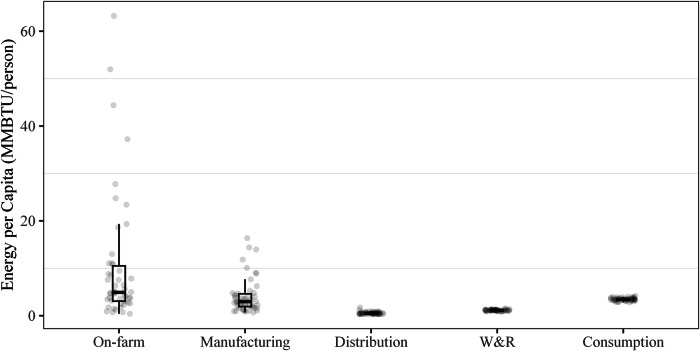
Box plots showing the spread of per capita site-energy consumption for each stage of the 2016 U.S. FSC.

This analysis shows that, at state level (shown in Fig. 11), the energy consumption and GHG emissions for the on-farm production, food manufacturing, and food distribution stage mainly aggregate in upper-Midwest states, California, and Texas, which are the major suppliers of agricultural materials and food products of the U.S. human food. In addition, the energy consumption and GHG emissions for the W&R and consumption stages are directly related to the population in each state. Given that the allocation of national-level data was done via food expenditures, this is not surprising (as described in Supplementary Note 8). Simple correlation statistics were run for each stage, comparing state-level energy use and population. While on-farm and manufacturing are very poorly correlated (*R*^2^ values of about 0.4 and 0.5, respectively), distribution had a moderately strong correlation (*R*^2^ of 0.8), and W&R and consumption both had very high correlations (*R*^2^ of about 0.99 for both). Additionally, the range of energy consumption per capita at the final three stages of the FSC was around 1 MMBTU per person, showing a very low per capita energy use variation between states (also shown in the box plots in Fig. 12). This implies little regional difference due to the energy Americans use to store and prepare food. This may be caused by a baseload effect from the high use of refrigeration (e.g., 380 TBTU compared to 70 TBTU for freezers and 350 TBTU for cooking for all U.S. households) or the high consumption of food from food services, where refrigeration use and preparation are even more standardized. Conversely, energy use and emissions in the on-farm and manufacturing stages are more influenced by the specific commodities produced in those states.

To explore this further, the energy and GHG intensity for each food commodity and state combination for the on-farm and manufacturing stages were estimated and compared to the national average for each commodity. Overall, states with lower production exhibited higher-than-average intensities, while the top 10 producing states tended to use less total energy or have fewer total emissions. Some commodity combinations displayed more variability than others. Therefore, the coefficient of variation (or relative standard deviation—the ratio of the standard deviation to the mean) was calculated for each category (Table 1).

**Table 1:** Coefficient of variation for state-level energy and emission intensities for on-farm and manufacturing commodities

		Animal products	Dairy products	Fruit, vegetables, nuts products	Grain, oil products	Sugar, confectionary products
On-farm	Site energy	3.3	1.4	0.79	2.1	0.49
	Primary energy	3.3	1.4	0.80	2.0	0.51
	GHG emissions	3.6	1.4	3.6	2.6	0.88
Manufacturing	Site energy	0.37	0.65	0.46	0.72	0.96
	Primary energy	0.42	0.70	0.44	0.76	0.82
	GHG emissions	0.60	0.72	0.42	0.75	0.58

**Fig. 13:**
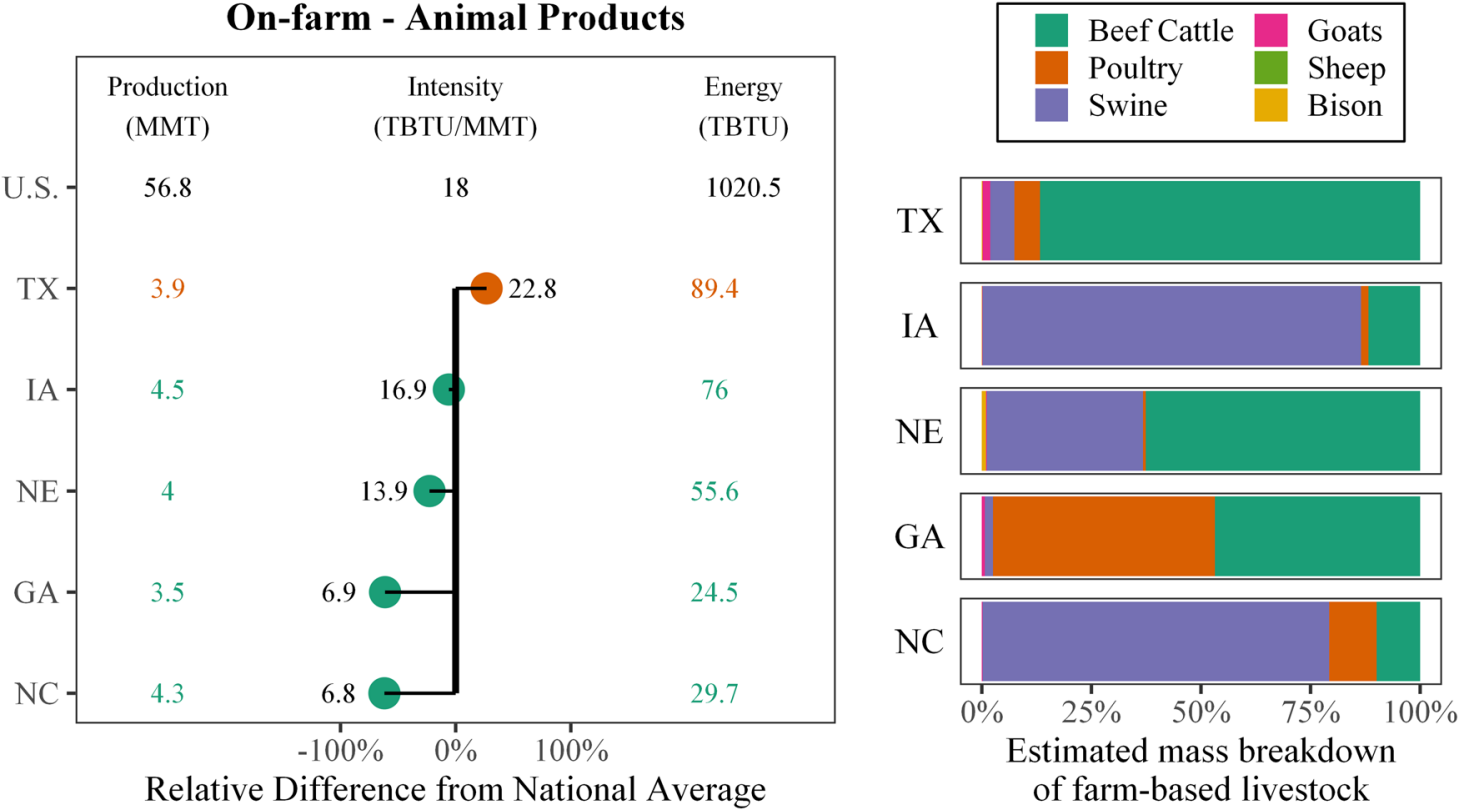
The 2016 on-site energy (TBTU), production (MMT), and intensity (TBTU/MMT) of the top five producing states for on-farm animal production (left) alongside the state’s animal products breakdown (right).

Overall, on-farm production energy use and GHG emissions had higher scatter than manufacturing, with most categories’ standard deviations greater than the mean (Table 1). The manufacturing of products is likely to vary less than farming practices for different products. For example, energy demands for raising cattle are much higher than poultry, but the process for freezing them is not. On-farm animal products’ energy use and GHG emissions had two of the highest scatter metrics. This is likely caused by the large variation in products and farming practices in the commodity category rather than any specific inefficiencies in some states. The commodity “animal products” includes beef cattle and swine as well as other lower-intensity products (e.g., poultry, seafood), leading to the wide variation in product intensities. This may have also contributed to Texas’s abnormally high intensity, despite it being a high-volume producer. Figure 13 illustrates the on-site energy intensity for the top five producing states for animal products, alongside their approximate relative breakdown of livestock. Here, seafood is not included in breakdown. Texas is predominantly represented by beef cattle, whereas the other top states have a higher percentage of swine and poultry. Similarly, grain and oil products have a high scatter with a wide range of products (e.g., corn, wheat, millet, sunflowers, cotton, soy, canola, and rice on the farm and countless products from manufacturing). While on-farm sugar generally consists of sugar beets and sugar cane, there are substantial differences in the farming of the two commodities^20^, and the low production of both likely contributed to the higher scatter. Like grain and oil product manufacturing, sugar and confectionary products high scatter in manufacturing can be contributed to the extremely wide range of products (e.g., sugar compared to candies).

Some of the differences in the state-level analysis may be caused by the allocation of data that are only available at the national level (e.g., crop and animal direct emissions, renewable energy use) and some assumptions in the analysis. For example, on-farm fruit, vegetables, and nut products also have a very high scatter for GHG emissions. As there is not a high level of scatter in site or primary energy use, the extremity in the emissions variation is likely caused by the allocation of crop-based emissions (see Supplementary Note 3) and not inherent in the states themselves. While the state-level data in this dataset can be used to help understand the U.S. food sector’s energy use and GHG emissions, care must be taken about conclusions drawn at the state level.

## Discussion

This study created a database detailing the U.S. domestic energy consumption and GHG emissions along the FSC by food commodity groups, FSC stages, and energy sources at the state level. This database can be utilized as a baseline for identifying the most impactful FSC stages or food commodities of energy use and emissions and designing efficient strategies toward net-zero emissions in the U.S. food sector. This section discusses the opportunities and strategies for energy use and GHG emissions reduction along the U.S. FSC based on the findings in this analysis and the database.

The database developed from the models created by this study compares well with others (e.g., FAOSTAT and EDGAR) at an aggregated level. Discrepancies arise primarily due to differences in boundary definitions of stages (e.g., the inclusion of fertilizer and pesticide manufacturing within “on-farm” rather than as a separate stage) and the source data used. For example, when looking at the on-farm production stage, the U.S. animal emissions, along with on-farm energy consumption and GHG emissions estimated by FAOSTAT, closely align with the estimates in this study. However, because this analysis includes emissions from soil management under crop emissions, the crop emissions in this study are significantly higher than those estimated by FAOSTAT. Additionally, the emissions from fertilizer and pesticide manufacturing estimated by this study differ markedly from those estimated by FAOSTAT. This discrepancy arises primarily from the differing methodologies employed. Examining the datasets more closely, energy consumption and GHG emissions from food processing and the transportation of raw food materials and final products closely match those reported by FAOSTAT. When it comes to W&R and Consumption, discrepancies arise again due to different methods and datasets used. Discrepancies also occur in food waste management, mainly due to differing definitions of this stage. For instance, FAOSTAT’s food waste management includes the landfilling of food waste in municipal solid waste, food waste in wastewater treatment systems, and incineration of food packaging. In contrast, this study does not include the management of food packaging and considers food waste management across the entire U.S. FSC and nine possible food waste management pathways. Additionally, this study considers the potential offsets in energy consumption and GHG emissions. A detailed comparison between this study and FAOSTAT is available in Supplemental Note 11.

Industrial electrification paired with a greener grid has been shown to potentially be a very powerful tool for decarbonization^21,22^. However, the U.S. electrical grid is currently less efficient in terms of primary energy than on-site fuel consumption (e.g., natural gas furnaces and boilers), and this is especially true for the FSC. While the on-farm, manufacturing, and distribution stages use little to no electricity, it is the main energy source for the W&R and consumption stages. This study estimated that the FSC accrued 2461 TBTU of electricity generation, transmission, and distribution losses in 2016, requiring 7118 TBTU of primary energy for only 4657 TBTU of site energy use. Food disposal activities that are not accounted for in the FSC add additional 144 TBTU of site energy but only 92 TBTU of primary energy because several of the disposal methods generating electricity and offsetting the grid’s low efficiency. In addition, the U.S. FSC generated 407 MMT CO_2e_ from energy consumption, with 56% of the energy-related GHGs from electricity production, indicating a greener electricity generation system is necessary for the United States’ net zero goals. The 2023 Annual Energy report^23^ by the EIA expects a shift in electricity production toward renewable sources, with 2050’s solar and wind capacity increasing by 1650% and 250%, respectively, compared to 2020. This shift will decrease the U.S. average emissions factor from around 830 kg CO_2e_ in 2020 to nearly 300 kg CO_2e_ in 2050. Additionally, with a greener grid, the electrification of farm equipment, manufacturing, and transportation would be a viable pathway to GHG reduction. The database generated by this study breaks down the site and primary energy consumptions of the FSC such that further detailed analysis into the impacts of changes to the production equipment (e.g., via electrification or improved efficiency) or changes to the U.S. electricity production portfolio are quantifiable. The database includes state-level breakdowns with differences in the electrical generation mix, making it easier for futures studies to explore more localized changes of grid mix for the FSC in that area.

Among all the FSC stages, on-farm production is the largest energy consumer and source of GHG emissions. The relatively lower emissions from fuels at this stage indicate that reducing direct energy consumption or switching to better energy sources (e.g., away from electricity now or towards electrification with a green grid) may not significantly impact on-farm GHG emissions reduction, though the clean energy transition would still be beneficial in a zero-carbon economy. A large portion of on-farm energy consumption is caused by raising animals (i.e., meat, poultry, eggs, dairy, seafood), with the model estimating nearly 1320 TBTU consumed on-site, requiring over 1530 TBTU of primary energy and, including non-energy emissions, producing nearly 510 MMT CO_2e_. There are several ways to reduce the energy demand and emissions of animal-based products. The farming, chemicals, transportation, and manufacturing of animal feed are large contributors to the commodities’ impacts (around 650 TBTU, 49% of animal product site energy). Any efforts to reduce its impact could be beneficial, such as less chemicals applied to feed, more grazing-based feeding practices, more FSC FLW diverted to animal feed. Conversely, the bigger impact on GHG emissions might not be with the production of feed, but with which feed is used. Enteric fermentation alone contributes a substantial quantity of GHGs (over 170 MMT CO_2e_)^24^, and changing the animals’ diets can have a substantial impact on the methane produced by digestion^25,26^. The high impact of animal products suggests that dietary change (e.g., consuming less meat and more alternative protein sources - e.g., seafood, nuts, and soy, or even just a higher shift from beef to chicken) could be a viable pathway to GHG reduction, while also keeping more in line with U.S. Department of Agriculture (USDA) and U.S. Department of Health and Human Services (DHHS) guidelines^27^. Any future studies using this dataset to estimate the effects of diet change on the U.S. FSC would need to account for any increases in other commodities to maintain a healthy diet. It is well known that U.S. households eat more than the recommended amount of most food groups. Following the suggested caloric intake and healthy diet recommendations would likely reduce the demand of each food commodity group and therefore decrease the overall U.S. food demand, waste, and related energy consumption and GHG emissions.

Another common suggestion to reduce the FSC’s environmental impact is optimizing the U.S. food distribution system. According to this study, which assumes each food manufacturer would serve all the U.S. states, the current food distribution system (i.e., manufacturers to W&R) only contributes 3.7% and 2.4% of the FSC’s site and primary energy (172 TBTU), and 1.4% of GHG emissions (13.2 MMT CO_2e_). However, by pairing the food suppliers and food consumers to minimize the total food-miles of each food commodity, the food distribution energy consumption and GHG emissions are reduced by about 7% (to 160 TBTU and 11.8 MMT CO_2e_, Supplementary Note 6). In addition, the optimized food distribution system can reduce transportation time, thereby increasing products’ shelf-life at the retail and consumption stages, especially for perishable food items. Correspondingly, this can reduce the amount of food discarded at households and W&R due to expiration by 6 MMT, which in turn would reduce the food demand and thus the FSC energy by an additional 1.7% and GHG emissions by 1.8% (following the methodology outlined in the DOE Sustainable Manufacturing and Circular Economy Report, Chapter 5)^28^. This study showed that the energetic and global warming potential cost of food waste is low, so shifting FLW disposal to more circular or energy-producing methods would benefit the overall FSC. Any circularity of wasted food back to the FSC would reduce the need to generate food from the very beginning. For example, more food donation would reduce the overall food demand. Similarly, more food waste could be used in animal feed manufacturing, reducing the need to grow crops for feed. However, a lot of FLW is not fit for either human consumption (e.g., bones, organs, or expired food) or even animal feed (e.g., sugars, processed foods). For such FLW, shifting away from landfill and incineration (even those with energy recovery) towards AD, composting and other methods could have a positive impact on the FSC energy consumption.

Reducing food waste generation is the best option to reduce the impact of FLW disposal, as it also reduces the amount of food that is necessary to grow and thus impacts the overall FSC. According to Dong et al.^11^, the U.S. provided nearly 900 MMT of agricultural materials in 2016, while only about 130 MMT of food products were consumed in the U.S. and about 420 MMT were used for other purposes (e.g., international trade, animal feed production), leaving 335 MMT of FLW managed through multiple pathways (excluding food donation, as it is consumed). As mentioned above, most of the U.S. FLW generation occurs at the W&R and consumption stages (21%). Furthermore, most of FLW generated at earlier stages is recycled in some way (91%). This has led to the USDA and U.S. EPA creating a nationwide goal of reducing food waste by 50%, focused on the W&R and consumption stages^29^. This study estimated that if the U.S. were to achieve that goal, the U.S. FSC’s site energy consumption would be reduced by 17% (792 TBTU) and its GHG emissions would be reduced by 16% (155 MMT). The impacts of the identified strategies are estimated with generalized assumptions and using the database created for this study. Future work by the authors or others can expand into detailed analyses to understand the benefits and impacts of each proposed strategy. Additionally, further work can be done to identify stage- and regional-specific strategies and goals, as well as other more substantial ways to change the environmental impact of the FSC (e.g., better energy profile by state or region, decentralized food supply).

The state-level analysis shows that there are substantial differences in the energy intensities of food production (i.e., on-farm activities and manufacturing) between states but little differences in per capita energy consumption in the consumer-facing stages. This is most prominent in commodity categories with significant differences in products; for example—there is a large difference in approximate energy intensities for raising beef compared to other animals (high variation in state-to-state energy intensity, correlating to different product mixes), but little difference in manufacturing. While this dataset can be used to draw some conclusions regarding state-level energy use in the FSC, caution should be taken. In many instances, the allocation to the state level or allocations within the state level (allocating impact to different commodities or fuels) was done based on regional averages or other assumptions detailed more in the Supplementary Notes. Overall, the more aggregation of the dataset, the more confidence in the conclusions drawn from it, as most assumptions were used to disaggregate the data rather than in the overall data generation. However, this dataset is provided in its fully disaggregated form to enable stakeholders to explore the data comprehensively, enabling them to reaggregate it as needed to address a variety of research questions.

**Fig. 14:**
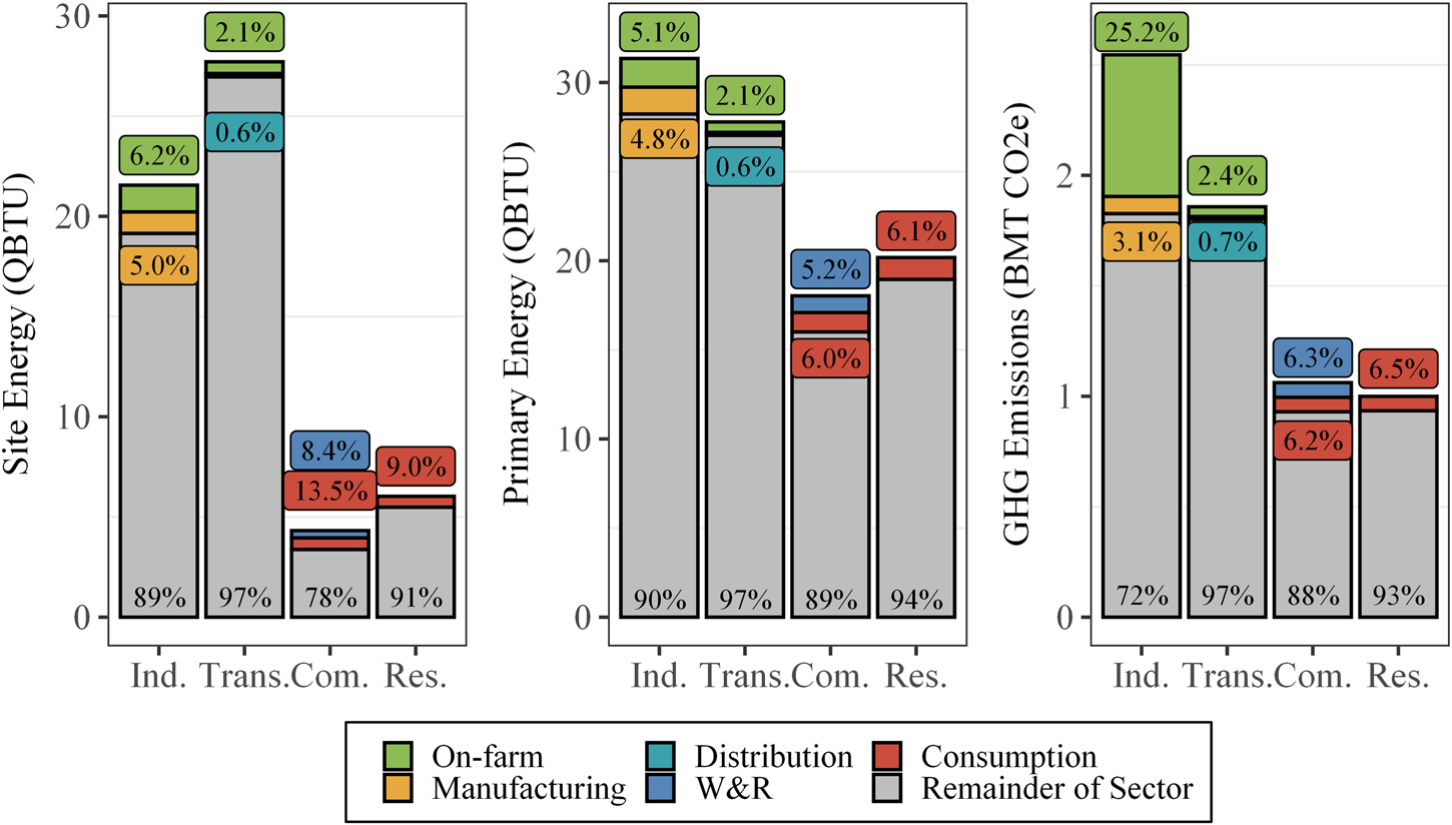
The U.S. FSC as part of the total U.S. energy use and GHG emissions^56^.

Overall, this study estimates that the U.S. FSC consumed 4660 TBTU of the site energy or 7130 TBTU of primary energy, generating 970 MMT of GHG emissions in 2016. This represents a substantial portion of the total 2016 U.S. energy consumption (7.8% site, 7.3% primary) and GHG emissions (15.0%). An unofficial rule of thumb has been that the FSC makes up 10% of the industrial (on-farm and manufacturing), commercial (W&R and food services), and residential sectors. This study partially supports this rough estimation, though on-site commercial is closer to 20%, (see Fig. 14), and the FSC in the transportation sector was shown to be less than 3%.

Finally, this work provides a compressive database illustrating the energy consumption and GHG emissions along the U.S. FSC by combining different large data sources with a breakdown into the primary U.S. food commodities and different FSC stages at the state level. The dataset provides the starting point for potential case studies that expand the discussion around the energy and GHG impacts of the U.S. FSC. Additionally, the methodology outlined here and in the Supplementary Notes and the model, available on GitHub^14^, provides a clear method of reproduction as new data is generated by the various sources.

## Methods

This study estimated the energy use and GHG emissions across the U.S. FSC using several sources from the USDA, EIA, EPA, and other literature sources. While some data were collected for individual commodity at the state level for individual fuel sources, in many cases, the data had to be estimated by merging various data sources, detailed below and in Supplementary Notes2–9. This study estimated the on-site energy consumption and the primary energy needed to produce that energy (matching sources like the EIA), and the GHG emissions of FSC. The inclusion of primary energy and its associated GHG emissions expands the systems boundaries to include the production of grid electricity that are used directly by the farm or indirectly by farm-related manufacturing (e.g., pesticides) from natural gas, coal, petroleum, nuclear, and several renewable sources. State-level data on electricity generation (e.g., generation and emissions by fuel sources, estimated losses) from the EIA were used to generate the primary energy and GHG emission data for electricity use. The data sources and method for converting grid electricity to primary energy consumption and GHG emissions for each FSC stage can be found in Supplementary Note 2. Emissions from direct fuel combustion were estimated using the EPA’s emission factors^30^, and refrigerant fugitive emissions were estimated based on national estimates^31^.

### On-farm production

The on-farm fuel and electricity expenditures by state or region, fuel type, and commodity were utilized and combined with EIA fuel and electricity prices by state, to estimate direct fuel and electricity use. Fuel expenditures at the state–fuel–commodity level (e.g., the amount of petroleum fuels needed for dairy production in Minnesota) were estimated by combining two major datasets: total farm fuel expenditures by state and commodity and fuel expenditure by region and type of fuel^32^. Once approximate fuel expenditure by state, commodity and fuel type was estimated, the state-level fuel prices from the same time period were used to transform the data to fuel use^33–36^. Like fuel, electricity expenditure was estimated based on on-farm utility^37^ and water^38^ use provided by USDA and then transformed to electricity use with state-level electricity prices^16^. The energy generated and used on-site for on-farm-based renewable energies was also estimated by combining the number of renewable energy operations on farms tracked by USDA^39^ with the EIA Annual Energy Outlook’s estimate for agricultural renewable energy use^23^. The considered renewal energy operations include biodiesel, ethanol, methane digesters, small hydro systems, solar panels, and wind turbines. More details about data source selection, data transformation, and filling missing data points to estimate on-farm direct energy consumption are described in Supplementary Note 3.

In addition to energy expenditure data, the USDA collects and provides data on fertilizer and pesticide application for different crops across each state^40^. The application rate was calculated and averaged for each crop in each state and combined with the production energy intensity for the agricultural chemicals^41,42^. Additionally, the production of ammonia and phosphoric acid for fertilizers have associated process emissions that were applied to each state and commodity by the application data^24^. More details about such calculations are available in Supplementary Note 4. Finally, the energy demand for animal feed manufacturing is discussed in “Methods: Food Manufacturing” about human food manufacturing and in more detail in Supplementary Note 5, as they required the same data sources and calculations. Similarly, the energy consumption for transportation of agricultural materials to manufacturing facilities was calculated in the same way for the transportation from manufacturing facilities to W&R locations, which is described in “Methods: Distribution”.

For all stages, the GHG emissions from the combustion of fuels and generation of electricity were estimated. However, the on-farm stage also includes several other emission sources. In addition to GHG emissions from the production of agricultural chemicals and animal feed, this analysis includes several crop-based (e.g., rice methane, soil management, field burning) and animal-based (i.e., enteric fermentation, manure management) emission sources. The U.S. EPA Annual Inventory of U.S. Greenhouse Gas Emissions and Sinks, Chapter 5^24^ provided national-level emissions, which were allocated to the states via relative sizes of field cropland and animals raised.

### Food manufacturing

The EIA’s 2014 and 2018 MECS were used to estimate the relative fraction of each energy type used for the available commodity-region combinations^19,43,44^. For missing commodity groups, this study used the difference between the total food data and the sums of the specified commodities’ data. The fraction was then combined with a database of all U.S. food manufacturing sites with 4-digit NAICS codes^45^ and the regression equations that relate sales to electricity and natural gas use based on the Industrial Assessment Center Database of assessments^46^ to estimate the distribution of the MECS data across the regions. Fugitive emissions from industrial refrigeration were estimated by distributing the United Nations Climate Change GHG Inventory Data estimate^31^ (9.00 MMT CO_2e_ of emissions in 2016) to different food commodities and states based on the MECS data allocation of process cooling energy use^47^. According to the USDA, approximately 2.3% of food sales are directly from farmers to retail, institutions, or food hubs, and 0.7% are directly from farmers to end consumers^48^. Therefore, the direct food sales from farmers without manufacturing were deemed negligible. In this study, all food products, including fresh vegetables and fruits, are assumed to go through food manufacturers before reaching the U.S. food market. The details of data source selection, data processing, and assumptions for the manufacturing stage can be found in Supplementary Note 7.

### Distribution

The distribution stage only covers the transportation from manufacturers to W&R. Even though transportation from farms to manufacturers is considered part of the on-farm stage, the calculation methodology for both is described here and detailed further in Supplementary Note 6. The energy consumption for human food distribution was calculated based on the estimation of food miles by food commodity groups across states and average fleet energy intensities (EI). The food miles were estimated by pairing the source (i.e., where agricultural materials or final products are produced) and destination (i.e., where agricultural materials or final products are required). For agricultural materials, it was assumed that the farm stage ships materials to manufacturers as close as possible (optimizing for minimum food-miles). For the manufacturing to W&R distribution stage, it was assumed that each food manufacturer provides food to every state, as most major brands are national brands. While this is likely an overestimation, the analysis showed that this is still the stage with the smallest contributions to energy use and GHG emissions among all FSC stages. The more optimized assumption is explored further in the “Discussion” section, and the methods are outlined in more detail in Supplementary Note 10. Energy use and emissions were assigned half to the origin state and half to the destination state.

This analysis assumes that both distribution stages, including farm-to-manufacturing and manufacturing-to-W&R, have the same fuel efficiency, and both rail and truck transport use only diesel fuel. The Transportation Databook^49^ provides average fleet EI of both long-haul trucks and rail, and Hwang et al.^50^ provided an average truck payload. Commodities requiring refrigeration during transport (i.e., dairy, fruits and vegetables, animal products, seafood) were assumed to require 20% more fuel than the U.S. average for trucks but no additional energy for rail^51^.

The GHG emissions from transportation-related fuel combustion and the refrigeration leaks from the refrigeration units (fugitive emissions) were estimated using the UN Climate Change GHG Inventory Data^31^ (5.89 MMT CO_2e_ of fugitive emissions for U.S. transport refrigeration in 2016) and the ratio of food mass flow (tonnes of food directed to each state) to the total mass of refrigerated goods transported across the U.S. in 2016.

### Wholesale and retail and consumption

The Commercial Buildings Energy Consumption Survey (CBECS) microdata^52^, USDA’s Cold Storage report^53^, and a few assumptions were used to estimate the energy intensities for U.S. dry and cold warehouses and the total storage space of U.S. warehouses. This was then used to calculate the energy consumed for food storage at the W&R stage. The detailed process can be found in Supplementary Note 8.

The total energy use for food sale, food services, and food-related residential energy in 2016 is reported by the EIA’s Annual Energy Outlook. The fuel split for these sectors is done using CBECS microdata and assumptions detailed in Supplementary Note 8. The total energy consumption data were allocated to the states using personal consumption expenditure data at retails and food services from the U.S. Bureau of Economic Analysis^54^ combined with Food at Home Monthly Area Prices data from the USDA to approximate total amount of food consumed^55^. This study assumes that energy intensities for food storage and preparation are approximately equal across the country.

GHG emissions from fugitive emissions for W&R and food services were estimated using the United Nation’s estimate for commercial fugitive emissions^31^ and allocated to states based on factors derived from the CBECS data. Please see Supplementary Note 8 for details. All of the residential fugitive emissions were assumed to be food-related, so the entire 1.28 MMT CO_2e_^31^ for 2016 was allocated to each state by retail expenditures, similar to the energy allocation methodology.

### FLW disposal

The energy and GHGs for FLW disposal that have not been already accounted for in the FSC were estimated using the mass flows estimated by Dong et al.^11^ and various intensity factors (see Supplementary Note 9). No explicit offsets were considered for this study other than electricity generation. Offsets such as fertilizer from AD or composting would already be accounted for by the total amount of fertilizer applied to crops, and offsets for bio-based industrial recycling applications are beyond the scope of this analysis.

## Supplementary information

Supplementary Information

## Data Availability

All the datasets adopted for this study are cited and detailed in Supplementary Notes. All the data sources are publicly available. All data are available at https://github.com/ORNL-Food-Supply-Chain/Energy_GHG.
