# Publisher Correction: Estimating energy consumption and GHG emissions in the U.S. food supply chain for net-zero

**DOI:** 10.1038/s41538-025-00425-8

**Published:** 2025-05-03

**Authors:** Kristina Armstrong, Wenquan Dong, Mingzhou Jin, Sachin Nimbalkar, Joe Cresko

**Affiliations:** 1 Manufacturing Energy Efficiency Research & Analysis, Oak Ridge National Laboratory, Oak Ridge, TN 37830 USA; 2 Ingram School of Engineering, Texas State University, San Marcos, TX 78666 USA; 3 Department of Industrial and Systems Engineering, The Institute for a Secure and Sustainable Environment, The University of Tennessee, Knoxville, TN 37996 USA; 4 Industrial Efficiency and Decarbonization Office, U.S. Department of Energy, Washington, DC 20585 USA

**Keywords:** Environmental impact, Agriculture, Economics

Correction to: *npj Science of Food* 10.1038/s41538-024-00346-y, published online 06 February 2025

In this article the wrong figure appeared as Fig. 4; the figure should have appeared as shown below.
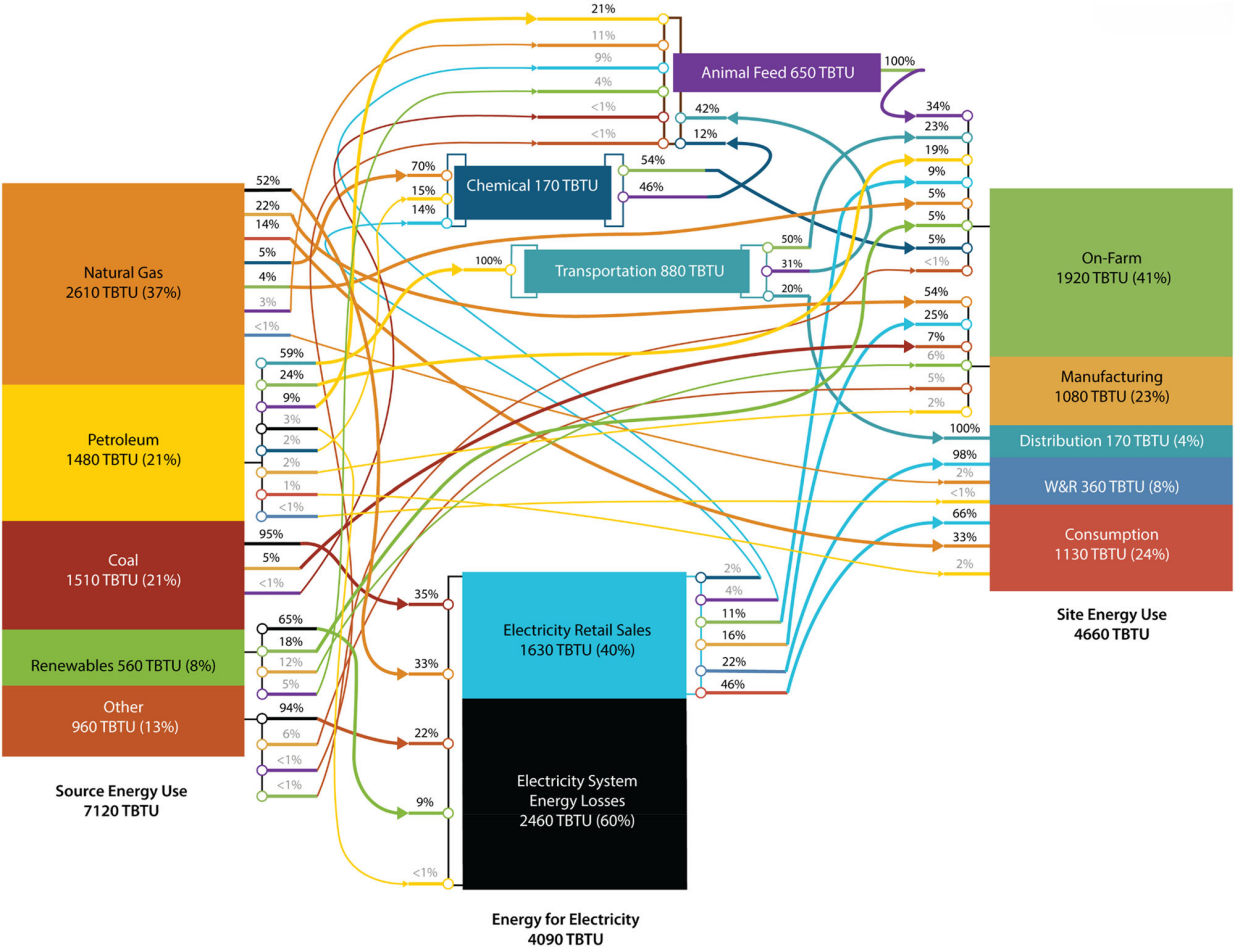


The original article has been corrected.

