# Supplementary material for: Estimating energy consumption and GHG emissions in the U.S. food supply chain for net-zero
Source: NPJ Sci Food. 2025 Feb 6;9:19. doi: 10.1038/s41538-024-00346-y (PMC11802779; doi:10.1038/s41538-024-00346-y)
Supplement: Supplementary file 1 — Supplementary Information [file 41538_2024_346_MOESM1_ESM.pdf]

Table of Contents

Supplementary Note 1 : Literature Review 2
Supplementary Note 2 : Primary Energy Consumption and GHG Emissions for Electricity Production and On-site Fuel Combustion 4
Supplementary Note 3 : On-farm Direct Energy Use and GHG Emissions 7
Supplementary Note 4 : On-farm Indirect Energy Use and GHG Emissions – Fertilizer and Pesticides 12
Supplementary Note 5 : On-farm Indirect Energy Use and GHG Emissions – Animal Feed 14
Supplementary Note 6 : Transportation of Agricultural Materials and Food Commodities 15
Supplementary Note 7 : Food Manufacturing Energy Consumption and GHG Emissions 18
Supplementary Note 8 : Energy Consumption and GHG Emissions at W&R and Consumption Stages 20
Supplementary Note 9 : Energy Consumption and GHG Emissions from FLW Management 22
Supplementary Note 10 : Optimized Food Distribution System 24

Supplementary Note 1: Literature Review

The quantification of the energy consumption and GHG emissions from food-related activities is well established at the global level. Rosenzweig et al. (2021) estimated that the global food system generates 21%–37% of the annual anthropogenic emissions, including 5-14% due to land-use change, across all economic activities [1]. Other studies such as Crippa et al. (2021) [2], Tubiello et al. (2021) [3] and Vermeulen et al. (2012) [4] support this range and all highlight the potential contribution of increasingly sustainable food systems to a net-zero emissions economy. However, these global-level studies lack a detailed energy consumption and GHG emissions analysis at the country and regional level, limiting their use in developing of regional or country specific strategies and targets [5,6]. Some global-level studies also break the GHG emissions and energy consumption down by countries. For instance, Xu et al. (2021)[7] estimated GHG emissions from plant- and animal-based foods around the year 2010 at the global- and regional level, revealing that the United States is one of the leading countries in GHG emissions from the production of animal-based foods. However, that study is primarily based on FAO's definition of the agri-food system, which, as discussed in Supplemental Note 11, differs from the FSC defined in the present study. Additionally, Xu et al. (2021) focus on GHG emissions at the global and regional levels, grouping all food commodities into plant- and animal-based categories, without providing a detailed analysis specific to the United States, such as state-level analysis by food commodity or fuel type. Both FAOSTAT [8] and European Commission's Emissions Database for Global Atmospheric Research (EDGAR) [9] maintain a database that tracks energy consumption and/or GHG emissions from agri-food systems by year, country, food commodities, and fuel types. However, again, they both follow the definition of FAO's agri-food system which has different coverage compared to the U.S. FSC defined in this study. Additionally, these two datasets primarily rely on global-level data, whereas this study, as detailed in Supplemental Note 11, relies primarily on U.S.-specific data maintained by various federal agencies.

Several U.S.-focused studies have estimated energy consumption and GHG emissions along the U.S. FSC by stage and food commodity group, revealing the impact of the U.S. food system. Efforts to quantify the U.S. food system energy consumption or GHG emissions date back to at least the 1970's [10], however, this study focuses on more recent efforts. Canning et al. (2010) conducted an input-output material flow analysis and estimated the energy consumption along the U.S. FSC (from on-farm production to food loss and waste management) from 1997 to 2002 [11], based on two federal data sources (i.e., the Bureau of Economic Analysis Benchmark Input-Output tables and the Energy Information Administration's State Energy Data System). However, this study only considered the energy consumption for landfilling FLW that was included in the municipal solid waste (excluding all of the other FLW management practices), based on EPA's Advancing Sustainable Materials Management, which has been considered an

underestimation [12]. More recently, Pagani et al. (2020) and Vittuari et al. (2020) conducted a more detailed estimation of the FSC energy use between 2004 and 2015 by FSC stage and food commodity, but the impact of FLW management was still outside the system boundaries [13,14]. Cuéllar and Webber (2010) [15] and Birney et al. (2017) [16] also estimated the embedded energy of U.S. FLW, however, the authors adopted the energy intensities from multiple case studies, rather than calculating the total energy use along the U.S. FSC. Each of these studies have their own faults and virtues, providing a comprehensive dataset and framework for analyzing the U.S. food-related energy consumption. However, a large portion of GHG emissions from the U.S. FSC are not caused by energy consumption, but directly by the crops and animals (e.g., soil management, enteric fermentation, manure management, rice cultivation).

Bozeman et al. (2019) examined the environmental impacts of food demand among U.S. demographic groups [17] instead of geographic food-related ecological impacts. Canning et al. (2020) estimated resource consumption and GHG remissions for the U.S. food demand based on a 2007 dataset, but considered the FSC in its entirety, without providing data disaggregated by food commodity groups and states [18]. By applying the U.S. Environmental Protection Agency's (EPA) environmentally extended input-output model and the Bureau of Economic Analysis' input-output accounts, Read et al. (2020) estimated the GHG emissions and other environmental impacts of the U.S. food system and the benefits of halving the U.S. FLW but again, this study did not provide data on specific resource consumption and GHG emissions by FSC stage and food commodity and the impact of FLW management was outside their scope [19]. Additionally, the U.S. EPA provides an annual estimation of the U.S. GHG emissions for different industries, including the agricultural sector and other sectors within the FSC, but it does not provide any detailed information by location, food commodities, o FSC stages [20]. The U.S. EPA also recently conducted a comprehensive literature review of energy consumption and GHG emissions related to the U.S. FLW. This study combines several studies with different boundary conditions to estimate an energy and GHG analysis of U.S. FLW (along with water, land use, and other impact factors), but its boundaries does not include the full FSC and they do not attempt a state-level analysis [21]. Similarly, efforts such as Heller and Keolian (2015) [22], Heller et al. (2018) [23], Birney et al. (2017) [16], and Guo et al. (2020) [24] focused on the GHG emissions associated with FLW management by adopting coefficients for energy intensity derived from literature and estimates of FLW mass flow. However, again, these studies did not provide detailed information on energy consumption and GHG emissions along the entire U.S. FSC.

Overall, none of the existing studies provide a comprehensive and detailed accounting for the energy consumption and GHG emissions of the entire U.S. FSC (i.e., from cradle-to-grave and by food commodity groups). Moreover, these studies have all focused on the global- or national- level, with no

state or regional analysis being conducted. This study fills these gaps by creating a comprehensive dataset detailing the energy consumption and GHG emissions by the FSC stage, contributor (e.g., fuel, electricity, fertilizer), and food commodity group both at the national and state levels.

Supplementary Note 2: Primary Energy Consumption and GHG Emissions for Electricity Production and On-site Fuel Combustion

Site energy is important for helping energy users understand and reduce their use but does not provide the full life cycle picture. There are many factors that greatly increase the amount of energy required to produce electricity, specifically transmission and distribution (T&D) losses and generation inefficiencies. This study reports energy use as both site energy and primary energy (includes the fuel use required to produce electric power). For fuels used on-site and for electricity generation, the conversion to embodied energy was considered negligible, thus this is still not a full life cycle accounting.

The conversion of site-electricity use to primary energy was estimated at the state-level using detailed state-level data from the Energy Information Administration (EIA). This data relates to the net-generation by type of producer and energy source [25] and was used to create a electricity generation power profile for each state (Figure S 1– right). This was then used to estimate the energy to generate the 2016 electrical generation for the state, using 2016 heat rates for electricity (shown in Table S 1) [26], as shown in Figure S 1, bottom-left. Noncombustible renewable energy used a standard conversion of 3,412 BTU/kWh as no fuels are used to provide this energy. The T&D loss factors for each state were estimated using the state electricity profiles for 2016 [25], which provide state-level total estimations of electricity generation, direct use, exports, and losses. T&D loss factors range from 1.0197 (for Wyoming) to 1.0899 (for Maryland) with a national average of 1.052 (see Figure S 1, top-left). Additionally, the EIA provides data relating the U.S. electric power industry estimated emissions by state [1]. When combined with the T&D loss factors, this data facilitates the estimation of GHG emissions per kWh of electricity used on-site, by state. Together, these combined datasets provided a way to break down the kilowatts of on-site electricity use into primary energy and GHG emissions by source fuel for each state, rather than using a general national-level factor.

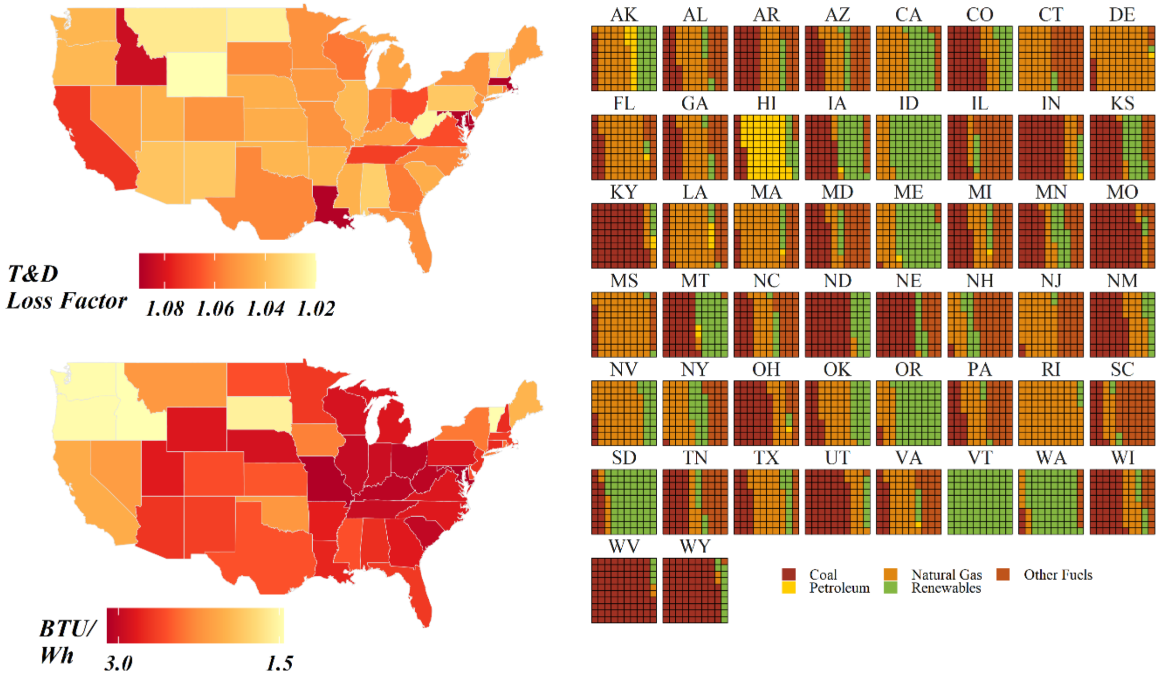

Figure S 1. Electrical Generation in the U.S. – Transmission and Distribution (T&D) Loss Factors for each state (top-left), Average energy required to generate electricity for each state (BTU/Wh, bottom-left), and source fuels for electricity generation for each state (right).

Table S 1. Energy input (Btu) required to produce a Wh of electricity (heat rate) for combustion and other non-renewable electricity sources. Non-combustion, renewable sources use a conversion of 3,412 Btu/Wh

Fuel / Electricity Source	***Heat Rate (Btu/Wh)***
Coal	10,493
Natural Gas	7,870
Nuclear	10,459
Other	9,232
Other Biomass	9,232
Other Gases	9,232
Petroleum	10,811
Pumped Storage	9,232
Wood and Wood Derived Fuels	9,232

GHG emissions for on-site fuels were estimated using the EPA's Emission Factors for Greenhouse Gas Inventories, shown in Table S 2[26,27]. These emission rates were converted to CO_{2e} using global warming potentials (GWP) of 25 for methane and 298 for N2O, as recommended by the EPA, consistent with the guidance of the United Nations (UN) Framework Convention on Climate Change (UNFCCC) [27].

Table S 2. GHG Emissions (kg CO2e / MMBTU) for energy sources used with the food supply chain.

Fuel / Electricity Source	***GHG Emission Rate (kg CO_2e / MMBTU)***
Coal	95.30
Natural Gas	53.11
Petroleum	75.30
Diesel	73.50
Gasoline	70.84
LP Gas	61.96
Other	53.11
Renewables (Manufacturing)	95.67

Supplementary Note 3: On-farm Direct Energy Use and GHG Emissions

The EIA's Annual Energy Outlook estimates 1,132.7 TBTU of energy was used for agricultural activities in 2016 [28]. However, this estimate includes forestry, crops grown for industrial use (e.g., clothing, paper, plastic, alcohol), horticulture, and tobacco. It also offers very little detail on energy use by product or at the state level, nor agricultural chemical use. The United States Department of Agriculture (USDA) offers multitudes of data on agricultural activities with a high-level detail disaggregation, but no direct data on energy use. Instead, for this analysis the on-farm expenditures by state or region, fuel type, and commodity were utilized and combined with EIA fuel and electricity prices by state, to estimate fuel and electricity use.

Energy Expenditures

Fuel expenditures were estimated by combining two major datasets: total farm fuel expenditures by state and commodity [29] and fuel expenditure by region and type of fuel [30]. The total farm fuel expenditures dataset was used to allocate the total amount of fuel spent in a state to the different commodities. For data entries covering multiple NAICS codes or NAICS codes with multiple commodity groups, more data manipulation was needed to allocate the fuel expenditures to the desired groups (see below). This was combined with the fuel breakdown by region dataset to estimate the expenditures for different fuel types, for each commodity and state, though the assumption that each state in a region had the same relative fuel use was necessary. The fuel type breakdown for Alaska and Hawaii was not provided in the datasets as they are not included in the Agricultural Resource Management Survey regions [31]. For these states, their state-level industrial energy fuel use breakdown was used instead [32,33] . In addition, Hawaii had a high "other fuel" use, which was investigated and determined to be petroleum-based jet fuel (i.e., petroleum) [32]. Next, the state-level fuel prices from the same time period as the data were used to transform the data to fuel use in physical units (e.g., gallons) then the fuels heat capacities were used to transform the data to energy units (e.g., TBTU) [34–37].

Similarly, the USDA datasets only provide utility use data instead of actual electricity use [38], fortunately, the USDA does provide estimates on state-level water expenditures [39], and it was assumed that electricity expenditures are the difference between the reported cost of utilities and water (excluding any other potential utilities such as telecommunications). Like with fuel, electricity use was transformed from expenditures to energy units using state-level electricity prices [40].

Commodity Group Allocation

In addition to the data transformations described above, additional steps were needed to allocate the data into the desired commodity groups. The USDA aggregates its data by North American Industry Classification System (NAICS) codes (see Table S 3), so several commodities were not easily

disentangled (e.g., grains and oil). Others were easier to separate: the commodity code for "Other Crop Farming" (1119) also included some other commodities that had specific data (11191 – Tobacco, 11192 – Cotton, 11193 & 11194 & 11198 – Sugarcane Farming & Hay Farming & Peanut/Sugar beet Farming). The category "Other" is composed of the difference between the total 1119 category and the sum of the three specific groups, along with Peanuts. The combined commodity "11193 & 11194 & 11198" (hay, sugar, and peanuts) was broken up using USDA estimates of sugar beet, sugar cane, peanut, and hay production [41]. Dong et al., (2022) [42] provided the fractions of each crop produced for non-human food use (e.g., animal feed and industrial use) which were used to allocate grain and oil energy to human food production, animal feed production, and other uses. Finally, animal feed energy use was reduced by removing a fraction estimated for pets (3.0%) and split between dairy cattle (18.1%) and other animal products (81.9%) based on approximate amount eaten [43]. This description is also presented in Table S 3.

Additionally, data points were missing (the provided entry was "(D)") for several data files with a high-level of specificity (e.g., fuel used per state by commodity; see Table S 4), this represents entries with only one reporting entity in the data, thus data was withheld to provide anonymity. To attempt to fill in missing data, the difference between the reported total energy use for a state and the sum of the reported energy use data (i.e., the "remainder") was allocated to all missing entities using the below methodology.

Table S 3. Food Commodity by NAICS Code with notes regarding allocation to designated food commodity groups.

NAICS code	***NAICS Designation***	***Food commodity group***
1111	Oilseed and Grain Farming	18.7% into Grain & Oil [42]<br>43.2% into Animal Feed [42]
1112	Vegetable and Melon Farming	Fruit, Vegetable & Nuts
1113	Fruit and Tree Nut Farming	Fruit, Vegetable & Nuts
1114	Greenhouse, Nursery, and Floriculture Production	Excluded
1119	Other Crop Farming	Calculated remainder of 1119 after removing 11191, 11192, 11193 & 11194 & 11198 – Allocated to Other
11191	Tobacco Farming	Excluded
11192	Cotton Farming	17% into Grain & Oil [44]
11193 & 11194 & 11199	Sugarcane Farming & Hay Farming & All Other Crop Farming (Sugar Beat, Peanuts)	Allocated based on weight of each product per state [45].<br>Sugarcane & Sugar Beat – Sugar<br>Hay – Animal Feed<br>Peanut – Other
112111	Beef Cattle Ranching & Farming	Meat & Poultry
112112	Cattle Feedlots	Meat & Poultry
11212	Dairy Cattle and Milk Production	Dairy
1122	Hog and Pig Farming	Meat & Poultry
1123	Poultry and Egg Production	Meat & Poultry
1124	Sheep and Goat Farming	Meat & Poultry
1125 & 1129	Aquaculture & Other Animal Production	Seafood

There were only four combinations of missing data: dairy and animal products, dairy and grain & oil, animal products and grain & oil, and just animal products (i.e., cattle feedlots and poultry & eggs). If dairy was one of the missing categories, the U.S. calculated average energy spend per cow (i.e., $123.58/cow for fuels) was multiplied by the number of dairy cows per state [43], and the remaining remainder was allocated to the other commodity. If grain & oil and meat products were both missing, each received half of the remainder, and if the two animal products were missing, the entire remainder went to animal products.

For example, Georgia had a "(D)" listed for both "fuel expenditures of cattle feedlots" and "fuel expenditures for grain & oil", meaning that these activities take place in the state, but there was only one entity reporting data. In this analysis, the remainder was calculated as the total agricultural fuel expenditure for the state of Georgia minus the sum of the agricultural fuel expenditures in Georgia for everything that was reported (beef/cattle ranches, hogs/pigs, poultry/eggs, sheep/goats, cotton, vegetables and melons, fruit trees and nuts, etc.). Half of the remainder was allocated to cattle feedlots (and thus "Animal Products" and the other half was allocated to Grain and Oil. Similarly, Louisiana had a "(D)" for both "fuel expenditures of cattle feedlots" and "fuel expenditures for dairies". The fuel expenditures for dairies were calculated based on the number of dairy cows in Louisiana and the U.S. average fuel energy spend per cow and the fuel expenditure for cattle feedlots was calculated as the remainder minus the calculated dairy expenditure.

Table S 4. Summary of missing data

Missing Data	***Fuel***	***Electricity***
Dairy and Animal Products	Delaware, Louisiana, Mississippi	Delaware, Georgia, Hawaii, Louisiana
Dairy and Grain & Oil	Alaska, Hawaii	
Animal Products and Grain & Oil	Connecticut, Georgia, Maine	
Animal Products		Arizona, Maine

Finally, 17% of the energy for producing cotton was allocated to the Grain & Oil group for cotton oil [44]. Apart from their use in previously mentioned calculations, Greenhouse, Nursery, and Floriculture and Tobacco Farming were excluded from this analysis.

On-farm On-site Renewable Energy

The renewable energies generated and used on-farm was also estimated. This does not include any large-scale generation to be sold to the grid, only that which is used on-site. The USDA tracks the number of renewable energy operations on farms by state and NAICS code (including biodiesel, ethanol, methane digesters, small hydro systems, solar panels, and wind turbines) [46], and this was combined with the EIA Annual Energy Outlook's estimate for agricultural renewable energy use (134.45 TBTU) [47] to allocate estimated renewable energy use to the different commodities and states.

On-farm GHG Emissions

GHG emissions from fuels were estimated based on their fuel type and GHG emissions from electricity use were based on the methodology outlined in Supplementary Note 2. GHG emissions from these renewables was estimated based on the type of renewable: renewable electricity generators (i.e., small hydro systems, solar panels, and wind turbines) were assumed to have no GHG emissions, biofuel systems (i.e., biodiesel, ethanol, methane digesters) were allotted GHG emissions in accordance to the EPA's emission factors (73.54 kg CO_2e/MMBTU, 68.5 kg CO_2e/MMBTU, and 52.34 kg CO_2e/MMBTU, respectively). This analysis unfortunately depends on the assumption that each renewable operation generates about the same amount of energy, though the re-aggregated U.S. total would remain consistent with the EIA analysis.

In addition to the GHG emissions from fuel combustion (both directly on the farm and indirectly via animal feed, fertilizer, and chemical production and electricity generation), there are direct GHG emissions from both crops and livestock. This study includes methane and nitrous oxide emissions from enteric fermentation and manure management of domestic livestock (excluding horses and other animals not within the FSC), rice cultivation, soil management and field burning and direct carbon dioxide emissions from liming and urea fertilization. GHG fluxes from land use and land use conversion were not included due to their estimated low impact and lack of data to allocate emissions to the states (i.e., emissions from cropland remaining cropland, -22.7 MMT CO_{2e}, and land converted to cropland, 54.1 MMT CO_{2e}) [48]. This analysis heavily utilized the EPA's Inventory of U.S. Greenhouse Gas Emissions and Sinks, Chapter 5 [48], and allocated the emissions to the different states via relative sizes of the commodities produced: field cropland (i.e., cropland devoted to rice for rice methanogenesis [49], total cropland for emissions from soil management [50], liming and urea fertilization, total pastureland for soil management of pasture [51], and total field cropland for field burning [52]) and animals raised (i.e., for all animal emissions [53]).

Supplementary Note 4: On-farm Indirect Energy Use and GHG Emissions – Fertilizer and Pesticides

In addition to energy expenditures data, the USDA collects and provides data on fertilizer and pesticide applications for different crops across each state. Unfortunately, this survey is not conducted for every crop and not all the crops are surveyed each year for each state. Many crops have not been surveyed since 1999 (i.e., almonds, hazelnuts, limes, pistachios, sunflowers, temples, walnuts) or 2000 (i.e., artichokes, beets, Brussel sprouts, eggplants, okra, radishes, sugar beets). The USDA chemical application data and USDA data on acres harvested (field crops, vegetables, and berries) [54] or bearing (fruit and nut trees)[55] was pulled for a number of years around 2016 (1999, 2000, 2012 – 2020) [56] and the application rate (mass per area) was calculated and averaged for each crop in each state. To estimate the chemical application for crops where there was no application data for a specific state, but there was a known quantity of land devoted to the crop, the U.S. average application rate for the crop and the amount of crop grown in the state were used. For cases where there was no application data for any state, but again the total crop area is known, the commodity group's U.S. average application rate was used. Finally, applications to hay were estimated assuming application rates found in literature [57,58] and tons of harvested hay [59]. This approach was validated by comparing to total U.S. consumption of fertilizers and lead to application rates slightly lower than the total consumption (9.8 MMT of nitrogen, 3.9 MMT of phosphate and 4.5 MMT potash applied compared to 11.75 MMT, 4.09 MMT, and 4.87 MMT in 2016) [60]. The discrepancy can be partially attributed to the exclusion of horticulture and landscaping applications. Applications estimated from this approach do closely match those obtained by Ludemann et al. (2022) [58], when excluding the "Residual" data (9.1 MMT, 3.9 MMT, and 4.2 MMT).

Next, steps were taken to remove applications to non-food uses of oils and grain crops and separate the crops used for animal feed, using ratios developed for Dong et al. (2021) [42]. Finally, the energy contained within the chemicals applied had to be determined. Fertilizers and pesticides are used extensively for the cultivation of food and animal feed crops. In 2019, the fertilizer use in the United States was estimated to be 12 million metric nutrient tonnes of nitrogen fertilizers, and around 4 million metric nutrient tonnes each of phosphate and potash fertilizers [55]. However, studies analyzing the energy and GHG emissions from the manufacturing of agricultural chemicals often rely on outdated process energy and emission data, with even the most recent studies utilizing energy data from the 1980s or early 1990s (Audsley et al. 2009 ; Gellings and Parmenter 2016 [61]). To match the boundaries of the rest of this analysis, only the energy to produce agricultural chemicals (or the energy required to produce the electricity used) is considered. Natural gas used as a feedstock and other embodied energies of the chemicals are not considered, as it was not considered for the total energy use nor in the electricity analysis. For fertilizers, this study uses modified values from West and Marland (2002) [62], but only considers 33% of the natural

gas use to be energy related (the remaining natural gas is assumed to be feedstock)[63]. Additionally, the electricity use is converted to on-site energy use using the factor provided within the same study[62]. For pesticides, older energy data was used to determine the energy consumption for individual chemicals (Audsley et al. 2009 [64]), while an updated market mix (EPA 2017 [65]) was used to calculate the weighted average energy intensity. The energy intensity by chemicals and fuel types is summarized in Table S 5.

Table S 5. Energy Intensity of agricultural chemicals by energy source

Chemical	***Electricity (MJ/kg)***	***Natural Gas (MJ/kg)***	***Petroleum (MJ/kg)***
Fungicide	5.8	32.6	6.20
Herbicide	4.97	70.1	8.00
Insecticide	28.8	24.3	6.90
Nitrogen	0.946	17.2	1.99
Phosphate	1.84	0.630	2.91
Potash	0.723	2.69	2.05

There is an additional 9.0 MMT and 1.0 MMT of process emissions from ammonia and phosphoric acid production that can be attributed to fertilizer production, though these values are further reduced when only considering food products grown for human or animal feed. This was allocated to different states and commodity groups based on the mass of applied fertilizer.

Supplementary Note 5: On-farm Indirect Energy Use and GHG Emissions – Animal Feed

While not called out explicitly above, the fuel, electricity, renewable energy, and chemicals used to grow animal feed is included within this analysis. Table S 2 shows that 43.2% of Grain & Oil (NAICS 1111) expenditures and all of Hay's expenditures (NAICS 11194) were allocated to animal feed and this is broken down further by specific grain and oil commodities in Table S 6. As the chemical data was provided for individual crops, additional data from the USDA Agricultural Statistics [66] were used to allocate the chemical use of specific grains and oils to animal feed. All other food was assumed to be intended for human food.

Table S 6. Fraction of grain & oil commodities grown for food, feed, or other uses.

	Food	***Feed***	***Other***
Wheat	32%	2%	66%
Rye	29%	12%	59%
Rice	40%	4%	56%
Corn	6%	36%	58%
Oats	32%	40%	28%
Barley	38%	12%	51%
Cotton	17%	0%	83%

The transportation of materials to animal feed manufacturing and the transportation of processed animal feed back to farms (discussed in Supplementary Note 6), as well as the manufacturing of animal feed (discussed in Supplementary Note 7) is allocated to the on-farm stage, though the computations are handled with other similar computations within the analysis.

Supplementary Note 6: Transportation of Agricultural Materials and Food Commodities

This study considers the transportation of agricultural materials from farm gates to animal feed and food manufacturers as on-farm production activities. Diesel fuel (petroleum) combustion is assumed to be the only energy source and the major contributor to GHG emissions at the distribution stages. Fugitive emissions from cold chain logistics are also included for this substage. The energy consumption for transportation of agricultural materials was calculated based on the estimation of food miles by food commodity groups across states.

Transportation of Agricultural Materials

The food miles by food commodity are estimated by pairing the source (e.g., where resources are produced) and destination (i.e., where resources are required) to minimize the total transportation distance. Let N denote the set of the 50 states in the U.S. and the District of Columbia. For each pair of different states $i, j \in N$, the transportation distance between i and j is defined by d_{ij} and estimated based on the geographic center of each state [67]. In addition, the transportation distance for agricultural materials within a state (i.e., d_{ii}) is assumed to be 500 miles [68]. Let F denote the set of food commodity groups with f as its index. Then for each food commodity f, the agricultural materials produced at state i, s_i^f, are derived from the Annual Agricultural Statistics [66] which is maintained by the U.S. Department of Agriculture (USDA). The total agricultural materials received by the U.S. food manufacturers for producing food commodity f is defined by D_f and has been estimated by Dong et al. (2022) [42]. The agricultural materials used by the U.S. animal feed manufacturers (D_A) are rederived from Annual Agricultural Statistics [66]. Please note that we assume no loss and waste during this transportation process. The agricultural materials required by manufacturers at state i are estimated based on D_f for $f \in F$, D_A, and manufacturing energy consumption for producing food commodities and animal feeds. For instance, given the total energy consumed by the U.S. manufactures for producing food commodity group f (i.e., TE_f) and the energy consumed by manufactures in state i for producing food commodity group f (i.e., E_i^f), the agricultural materials f shipped to state i is assumed as $\frac{D_f E_i^f}{TE_f}$. Then, the mass flow of agricultural materials from state i to j for manufacturing food product f (i.e., x_{ij}^f) can be obtained by solving the optimization model denoted by Equation (S1) -(S3).

$$\min \sum_{j \in N} \sum_{i \in N} d_{ij} x_{ij}^f \qquad (S1)$$

$$\sum_{i\in N} x_{ij}^{f} = \frac{D_f E_j^f}{TE_f}, \qquad j \in N \tag{S2}$$

$$\sum_{j\in N} x_{ij} \le s_i^f, i \in N \tag{S3}$$

$$x_{ij} \ge 0$$

The objective function (S1) minimizes the total food miles for delivering agricultural materials to manufacturers for each food commodity and animal feed processing. Constraints Set (S2) ensures that the demand for manufacturers at each state is satisfied. Constraints Set (S3) represents that the agricultural materials shipped from each state do not exceed the state's production capacity.

Based on the mass flow at the state level, the food miles for each food commodity are calculated with the assumptions that

1) Road transportation (i.e., truck) is used to deliver agricultural materials if the shipment distance to food manufacturers is within 950 miles, otherwise, railway transportation is adopted;
2) For shipment to animal feed manufacturers, only road transportation is considered.

Distribution of Food Commodities

As mentioned in the main text, it was assumed that each food manufacturer provides food to every state, as most major brands are national brands. The food commodity f produced and entered the W&R stage in the U.S. is defined by P_f and $W\&R_f$, respectively and were estimated by Dong et al. (2022) [42,68]. State level production capacity of product f is also determined by the state level manufacturing energy consumption (e.g., the food products f manufactured in state i can be expressed by $\frac{P_f E_i^f}{TE_f}$). In addition, this study assumes that the FLW generated during distribution is due to the rejections by wholesalers and retailers, and therefore the food products left the manufactures gate equals the products arrive at the next stage. Due to the assumption that food demand in each state is determined by the population level, we assume the food product f shipped from state i to j is $\frac{P_f E_i^f a_j}{TE_f A}$ where A and a_i are the population at the U.S. and state i [69].

To determine the transportation mode, we assume road transportation is adopted for shipment within 740 miles and railway transportation for the remaining goods [70]. Moreover, if railway transportation is adopted, 500 miles round trip of truck shipment is added to food miles due to the distance between the manufactures/wholesalers and retailers and railways stations.

Estimating Energy Use and GHG emissions of distribution

The above analysis provided state-to-state ton and ton-mile freight data. Each state was then allocated half the tonnage and ton-miles for freight from the state and half the tonnage and ton-miles for freight into the state. These data were uses to estimate the energy demand (ton-miles) and the refrigeration needs (tons) of transport.

The Transportation Databook provides average fleet EI of both heavy single-unit and combination trucks (31,335 Btu per vehicle-mile) and Class I freight railroad (299 Btu per ton-mile). The average payload of the truck distribution fleet was estimated using Hwang et al.'s [71,72] distribution of 2017 traffic volume for vehicle class 5 – 13 and the average payload per class. As the FSC uses refrigerated trucks to transport perishable commodities, it was assumed that these commodities (i.e., dairy, fruits and vegetables, animal products, seafood) required 20% more fuel than the U.S. average. The refrigeration unit within railway transportation was considered to be a negligible addition to total energy use and ignored [73].

The GHG emissions from transportation-related fuel combustion and the refrigeration leaks from the refrigeration units (fugitive emissions) were estimated. According to the UN Climate Change GHG Inventory Data [74], U.S. transport refrigeration (including non-food transportation) had 5.89 MMT CO_{2e} of fugitive emissions in 2016. The fugitive emissions for food and agricultural materials transportation were then estimated using the ratio of food mass flow (tonnes of food allocated to each state) and the total mass of refrigerated goods transported across the U.S. in 2016, assuming the same fugitive emission factors for different commodity groups.

Supplementary Note 7: Food Manufacturing Energy Consumption and GHG Emissions

Energy use in food-related manufacturing was estimated using data from the EIA's 2014 and 2018 Manufacturing Energy Consumption Surveys (MECS) [75,76] and the U.S. DOE's Industrial Assessment Center Database [77], and the MNI database . While the MECS data provides energy use data (e.g., NAICS codes 311 – Food manufacturing, 3112 – Grain and Oilseed Milling, 31131 – Sugar Manufacturing, 3114 – Fruit and Vegetable Preserving and Specialty Food, 3115 - Dairy Product, 3116 – Animal Slaughtering and Processing), several commodity groups are not explicitly provided; additionally, it only has regional-level data, not state-level data. This dataset was used as the high-level totals for each region-commodity group combination with missing commodity groups using the difference between the total food data and the sums of the specified commodities' data and the other datasets were utilized to help provide the state-level allocation.

The MNI database contains sales, employee counts, and production area data for every manufacturing site in the U.S. with their 6-level NAICS codes (sites that only provided a 3-digit code were further classified using the provided SIC code). Unfortunately, it does not include any energy cost or use data. To connect this database with energy, regression models were developed from sites in the IAC database. IACs have performed free energy assessments for small to medium manufacturing facilities since 1980, and collect data for each site about sales, employee counts, and production area, as well as electricity and natural gas use. The database was filtered for assessments conducted after the year 2000 for the 311 NAICS code. Regression equations on sales and employees (together and separate) were estimated for each 4-digit NAICS code. Additionally, the sugar and confectionary subsector (NAICS 3113) was separated into sugar manufacturing (NAICS 31131) and confectionary (NAICS 31133, 31134, 31135, represented below as "3113X") to match the MECS data. Unfortunately, while nearly all the regressions were statistically significant (p-values less than 0.05), very few had appropriate R^2 (shown in Figure S 2). Additionally, once applied to the MNI database, the total energy use of the sector was greatly overestimated (likely due to the low correlations, regressions from low-to-medium sites being applied to large scale sites, and the MNI database providing sites from 2020, not 2016). It was determined that as the goal was to allocate the previously established MECS data to unquantified NAICS and states, the regression-based energy data could be used to estimate the fraction of total energy use a site uses as a fraction of the total for that region-NAICS code combination. This fraction was then applied to the MECS data to allocate the high-level data, providing state-level data for each 4-digit NAICS code.

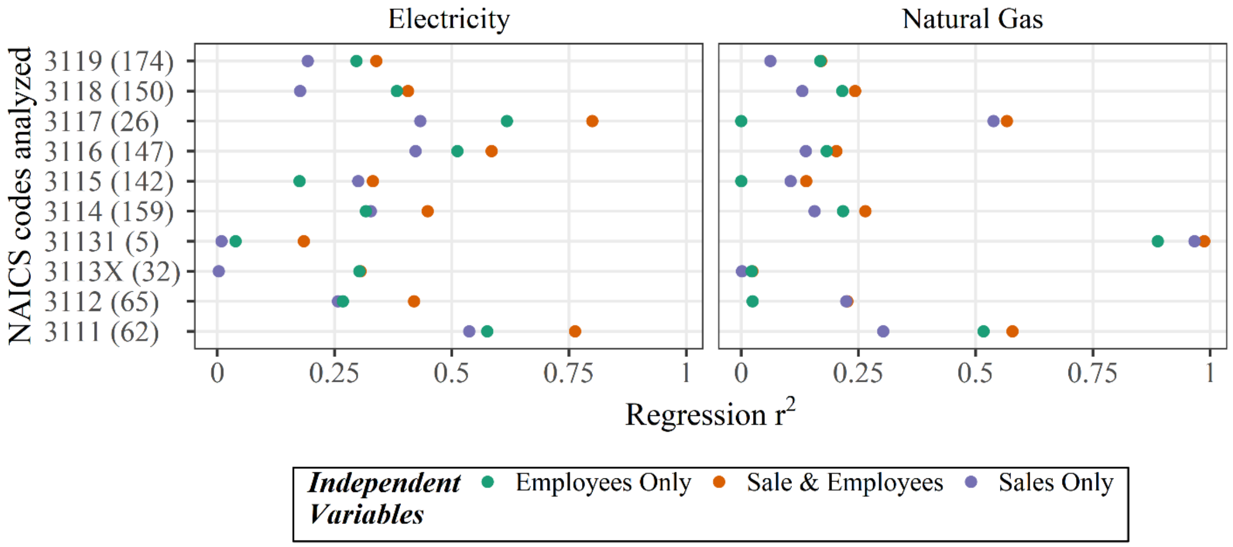

Figure S 2. Range of r^2 values for IAC electricity and natural gas regressions. The population size for each NAICS code is represented in parentheses with the value and all manufacturing within NAICS 3113 excepting 31131 is represented by "3113X".

Additionally, fugitive emissions from industrial refrigeration were included in the GHG analysis. According to the UN Climate Change GHG Inventory Data [74,78–81], industrial refrigeration had 9.00 MMT CO_{2e} of emissions in 2016. However, the UN Data does not provide a detailed breakdown by commodity or state-level and includes non-food related refrigeration. According to the MECS (Table 5.4)[82], the U.S. food manufacturers used 71 TBTU for process cooling. The MECS dataset also provides commodity-level data for several subsectors (i.e., Grain & Oil Milling; Sugar, Confectionary; Fruit, Vegetables, Nuts Products; Dairy Products; Animal Product Processing), some of the remaining commodities were assumed to be similar enough to other sectors to use the same process cooling ratio (e.g., seafood is similar to animal processing) and the difference between the total industrial process cooling energy and that of the known subsectors (i.e., "other") was split between the remainder. From there, the ratio of energy used for process cooling of the sector to the total U.S. manufacturing process cooling energy use was estimated, then applied to the total fugitive emissions to estimate the GHG from each subsector. Finally, to allocate the GHG emissions to each state, this was divided by the total electricity used by each sector and allocated to each state-subsector combination using the total electricity for the group (equation S4).

$$GHG\ Emissions_{State,Subsector} = Electricity_{State,Subsector} * \frac{GHG_{Ref.,Ind.}}{Electricity_{PC,Ind.}} * \frac{Electricity_{PC,Subsector}}{Electricity_{Subsector}} \qquad (S4)$$

Supplementary Note 8: Energy Consumption and GHG Emissions at W&R and Consumption Stages

The energy consumed for food storage at the W&R stage was estimated using the energy intensities for U.S. dry and cold warehouses and the total storage space of U.S. warehouses. The Commercial Buildings Energy Consumption Survey (CBECS) microdata provides approximate energy intensities for dry and cold warehouses for electricity, natural gas, and petroleum use by census region [83]. An estimate of the number and total cubic feet of public and private cold-storage warehouses in the U.S. and by state is detailed in USDA's Cold Storage report [84]. However, the public or private cold storage capacity data for some states are missing (if there is only one reporting entity in the state). To fill the data gap, the difference between the reported U.S. total cold storage (3.6 billion ft^3) and the sum of all the state-level data provided was calculated and distributed to the states missing a cold storage capacity by the number of warehouses in the state (assuming that all the warehouses are approximately equal size). In addition, the reported cold storage capacity is considered an underestimation, with only an estimated 78% of cold storage warehouses reported, [85] and no direct data on dry storage was found. The total U.S. cold and dry warehouse footprints were then estimated from this dataset with the following assumptions:

- Added 22% to each state's cold storage footprint [85]
- There is no dry public storage [85]
- 65% of private storage is dry, 35% is cold [85]
- The average U.S. warehouse height is 16.8 ft [85]

The EIA's Annual Energy Outlook for 2018 estimates the 2016 total delivered energy use for food sales (retail) to be 331.596 TBTU and the energy use for food services (consumption) to be 584.817 TBTU [86], with no details on source-level consumption. This analysis assumed that all the energy for food sales was electricity, however, natural gas is often used for food preparation in food services. The fuel split for food services was estimated using the CBECS microdata, by calculating the fraction of natural gas (45.4%) and electricity (54.3%) used in the food services sector; the remainder was fuel oils.

The food-related residential energy was also estimated using the 2018 AEO, by consolidating the energy for residential cooking, refrigeration, and freezers for a total of 545.27 TBTU of electricity, natural gas, and petroleum [87]. The data for all three of these substages was allocated to the states using personal expenditure data from the U.S. Bureau of Economic Analysis (personal consumption expenditure at retail and food services) combined with Food at Home Monthly Area Prices data from the USDA to approximate total amount of food consumed [88]. This study assumes that energy intensities for food storage and preparation are approximately equal across the country.

GHG emissions from fugitive emissions for W&R and food services was estimated using the UN's estimate for commercial fugitive emissions (45.46 MMT CO_{2e}) [74,89] and allocated to states based on factors derived from the CBECS data. The CBECS microdata includes estimates of energy used to refrigerate, so a fraction of energy used for refrigeration was derived for cold storage warehouses, food retail, and food services (PBAPLUS codes 10, 14, 15, 20, 32, 33, 34) for each census region. GHG Emissions from fugitive refrigerants were then estimated assuming that the ratio of GHG emissions and electricity used for refrigeration for given state and stage was equal to the ratio of GHG emissions and electricity used for refrigeration for the entire commercial sector (see the following equation).

$$GHG\ Emissions_{State,Stage} = f_{Refrigeration\ energy} * Electricity_{State,Stage} * \frac{GHG_{Commercial\ Refrigeration}}{Electricity_{Commercial\ Refrigeration}}$$

Supplementary Note 9: Energy Consumption and GHG Emissions from FLW Management

Dong et al. (2022) [42] estimated the FLW generated at each FSC stage and estimated the mass flow disposed of via several different methods (e.g., landfill, incineration, animal feed, donation). Using that work as a starting point, this study estimated the amount of energy is necessary to process the FLW via the relevant disposal method. The FLW mass data is currently only available at the national-level instead of state-level, thus this analysis is only at the national-level. As shown in Figure S 3, adapted from Dong et al. (2022) [42], the manufacturing stage is the largest contributor to FLW; however, most of it is remanufactured (137.5 MMT; i.e., used as animal feed, land application, composted, bio-feedstocks, or sent to a food donation center), and only 4.6 MMT is sent to landfill or incineration (none is sent to wastewater treatment). This is also the only stage that is known to contribute a substantial portion of its FLW to animal feed. The consumption stage is by far the largest contributor to wasted food (43.8 MMT, landfilled, incinerated, or sent to wastewater treatment), and is the only stage to utilize wastewater treatment as a disposal method.

Several FLW disposal methods were considered to already be a part of the established FSC. Preparation of donated food is accounted for in the consumption stage, as the energy data makes no distinction between food obtained at a grocery store and a food donation center/food bank. Similarly, the animal feed manufacturing stage analysis makes no distinction between feed stock from a farm and food waste from elsewhere in the FSC. Factors for transporting those food streams were added to the analysis.

The energy and GHGs for FLW not already accounted for in the FSC were estimated using the mass flows and various intensity factors. Energy intensities for food donation and animal feed manufacturing were estimated using the energy and GHG emissions calculated in this study and utilizing mass flows from Dong et al. (2022) [42] to estimate energy and GHG intensities. Energy and GHG emission intensities for other disposal methods, as well as for the transportation of donated food and animal feed were taken from other sources [90–93]. No explicit offsets were considered for this study other than electricity generation, as offsets such as fertilizer from AD or composting would already be accounted for by the total amount of fertilizer applied to crops and offsets for other manufacturing bio-based products are considered beyond the scope of this analysis. Figure S 4 shows the individual energy and GHG sources and offsets for each stage, with the net impact represented by the grey bar. This net impact value is the same shown in Figure 10 of the main analysis.

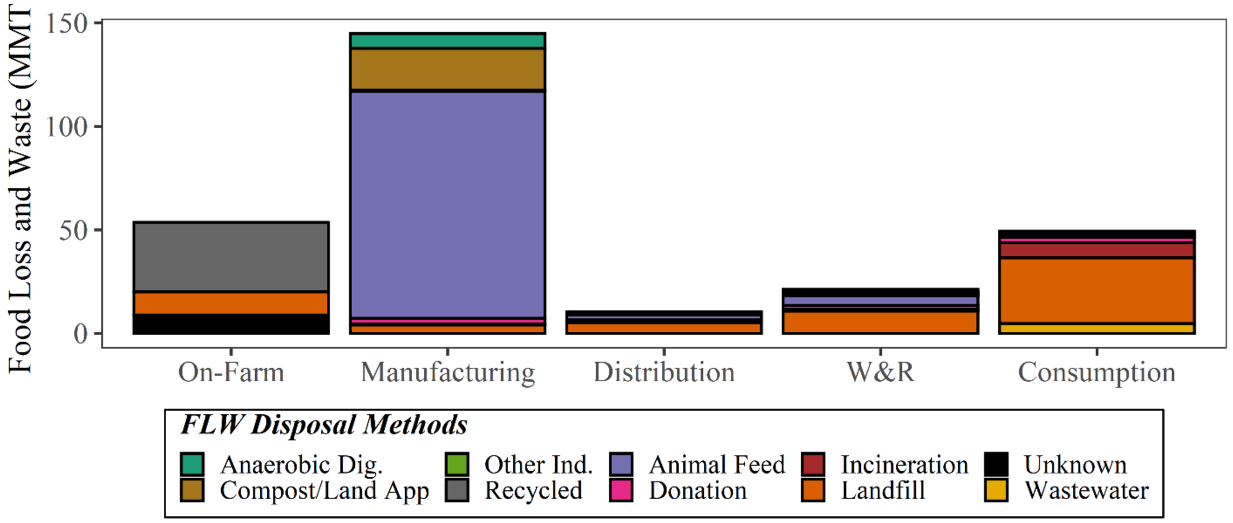

Figure S 3. FLW destinations, as described by Dong et al. (2022)[42]

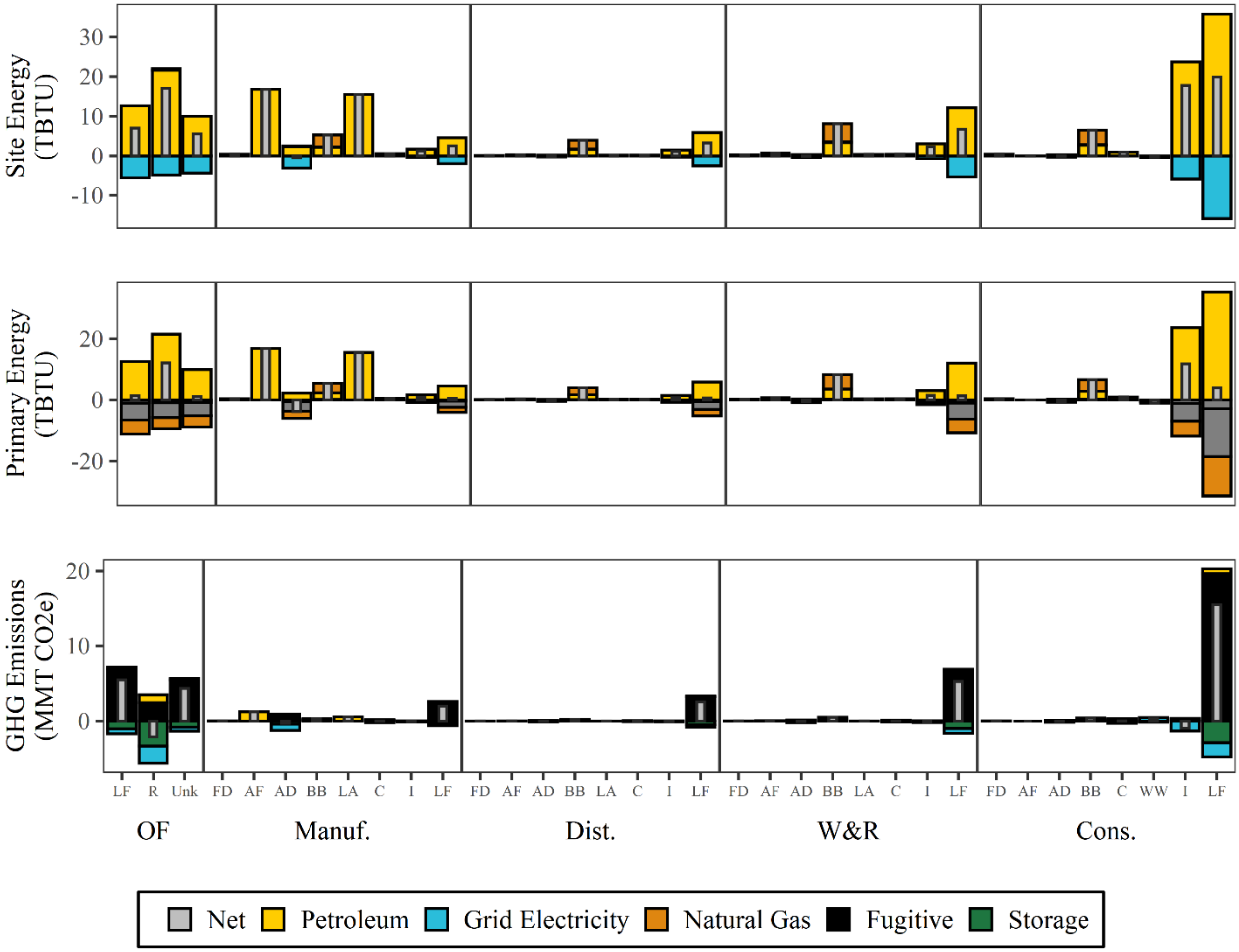

Figure S 4. Additional End of life site energy and GHG emissions for each EoL pathway (LF – landfill, R – recycled by unknown means, Unk – disposed of by unknown means, assumed to be landfill, FD – food donation, AF – animal feed, AD – anaerobic digestion, BB – bio-based industrial products, LA – land application, C – composting, I – incineration) in the 2016 U.S. FSC, broken down by contributor and stage (OF – on-farm, Manuf. – manufacturing, Dist – distribution, Cons. – consumption). The thin grey bar represents the net impact for the disposal method.

Supplementary Note 10: Optimized Food Distribution System

Same as the estimation for transportation from fields to manufacturers, the food distribution system can be optimized to minimizing food miles by ignoring the impact of food brands. The transportation distance between food manufactures in state i and wholesalers and retailers located j is defined by d'_{ij}, and $d'_{ij} = d_{ij}$ for $i, j \in N, i \neq j$. However, we assume $d'_{ii} = 450$ miles for all $i \in N$ [68]. Again, the food commodity f produced and entered the wholesale and retail stage in the U.S. is defined by P_f and $W\&R_f$, respectively and were estimated by Dong et al. (2022). State level production capacity of product f is also determined by the state level manufacturing energy consumption (e.g., the food products f produced in state i can be expressed by $\frac{P_f E_i^f}{TE_f}$). In addition, this study assumes that the FLW generated during distribution is due to the rejections by wholesalers and retailers, and therefore the food products left the manufactures gate equals to the products arrive at the next stage. With the assumption that food demand in each state is determined by the population level, we assume the food product f required by state i is $\frac{P_f a_i}{A}$ where A and a_i are the population at the U.S. and state i [69]. The mass flow (y_{ij}^f) at the state level is then obtained by solving the optimization model defined by Equations (S5) - (S7).

$$\min \sum_{j \in N} \sum_{i \in N} d'_{ij} y_{ij}^f \qquad (S5)$$

$$\sum_{i \in N} y_{ij}^f = \frac{P_f a_j}{A}, \qquad j \in N \qquad (S6)$$

$$\sum_{j \in N} y_{ij} \leq \frac{P_f E_j^f}{TE_f}, i \in N \qquad (S7)$$

$$x_{ij} \geq 0$$

To determine the transportation mode, we assume road transportation is adopted for shipments within 740 miles and railway transportation for the remaining goods [70]. Moreover, if railway transportation is adopted, 500 miles round trip by truck is added to food miles, accounting for the distance between the manufactures/wholesalers and retailers and railways stations.

Supplementary Note 11 : Comparison between This Analysis and FAOSTAT

This analysis compares well with FAOSTAT [8], a comprehensive database that provides global and national-level data on GHG emissions and energy use across various sectors, including the agrifood system in the United States. However, the agrifood systems defined by the FAO have different boundary scopes compared to the U.S. FSC defined and utilized in this study, leading to differences in the categorization of agrifood system/FSC activities and presented results. This and other high-level key differences are highlighted in Table S 7 at the end of the section (for example, this study does not account for emissions from land use change and the FAOSTAT data does not provide energy consumption from fertilizer and pesticide manufacturing).

While FAO categorizes activities under Farm Gate and Pre- & Post-Production, this study organizes them from a supply chain management perspective into six distinct FSC stages: on-farm production, manufacturing, distribution, W&R, consumption, and EoL management. By adopting this supply chain management perspective, the study not only facilitates comparisons of the U.S. FSC with those in other sectors but also underscores the importance of efficient supply chain management in achieving a sustainable U.S. FSC.

The Farm Gate category, as defined by FAO, includes emissions from crop and animal production, the application of fertilizer, organic soil management, and on-farm energy use. The Pre- & Post-Production category encompasses emissions from everything else related to the FSC: fertilizer and pesticide manufacturing, food processing, transport, food retail, household consumption, food storage (e.g., refrigeration), and food waste management.

In contrast, this study's on-farm production stage includes all activities within FAO's Farm Gate category (i.e., on-farm energy use, crop and animal emissions), as well as fertilizer and pesticide manufacturing and the transportation of food materials from the farm gate to food manufacturers, which FAO classifies under Pre- & Post-Production. Furthermore, the FSC in this study includes emissions from soil management and animal feed manufacturing—activities excluded from FAOSTAT.

Within the rest of the post-production category, the food manufacturing stage in this study aligns directly with FAO's definition of food processing, but W&R and consumption have some differences in scope. The W&R stage in this study includes energy consumption and emissions from food retail and wholesale stores, whereas FAOSTAT, although not explicitly stated, likely includes food services within the food retail category. Finally, the consumption stage in this study groups household consumption with food service activities.

While both FAOSTAT and this study include food waste management, their coverage differs substantially. FAOSTAT's food waste management considers the landfilling of food waste in municipal solid waste, wastewater treatment, and incineration of food packaging, making it very likely it only considers the disposal of waste from the more consumer facing sectors (e.g., retail, food services, and households). According to Dong et al. (2021) [42], while food waste in municipal solid food waste is mostly driven by the consumer stage, the other stages do produce substantial amounts of food waste that are handled through several different management pathways that all require additional energy inputs. Therefore, while this study does not include the management of food packaging, it does consider food waste management across the entire U.S. FSC with nine possible food waste management pathways. This study also considers the potential offsets from FLW management for energy consumption and GHG emissions.

Finally, while both datasets estimated emissions for most activities and stages, the FAOSTAT only reports on-site energy consumption and only for some of the categories. This study reports both on-site and primary energy consumption. A detailed comparison of FAOSTAT and the dataset created by this study is provided below.

Table S 7. Mapping U.S. FSC to Agri-food Systems and Comparing with FAOSTAT

FAO Definition			Agri-food systems / FSC activity	GHG [a] (MMT CO_{2e})		Energy Consumption (PJ)		This Study's Definition	
				FAOSTAT	This Analysis	FAOSTAT	This Analysis		
Agri-food Systems	Land Use and Change		LULUCF	NA [b]					
	Farm Gate	Crop Emissions	Direct crop emissions [c]	48	125	NA		On Farm Production	Food Supply Chain
		Animal Emissions	Direct animal emissions	247	248				
			Animal feed growth and manufacturing	Not Reported	213	Not Reported	687		
		On-farm Energy Use	On-farm direct energy use	69	56	825	776		
	Pre- & post- Production	Fertilizers Manufacturing<br>Pesticide Manufacturing	Fertilizer and pesticide manufacturing	22	9	Not Reported	99		
		Food Packaging<br>Food Processing	Food processing & packaging	102	81	1,168	1,136	Manufacturing	
		Food Transport	Transport – Farm gate to food manufacture	97	48	3[d]	641	On Farm Production	
			Transport – Food manufacture to end consumer					Distribution	
		Food Retail	Wholesale and Retail	50	132	1,430	1,000	W&R	
			Food Services					Consumption	
		Food Household Consumption	Household consumption	94	65	938	575		
		Solid Food Waste<br>Domestic Wastewater<br>Industrial Wastewater<br>Incineration	FLW management	47 [e]	70	Not Reported	144	EoL Management	

[a] Based on 100-year global warming potentials using the Intergovernmental Panel on Climate Change [IPCC] Fourth Assessment Report [AR4] values
[b] Emissions due to LULUCF are not included in this study and therefore, the table does not show FAOSTAT's data
[c] The crop emissions estimated in this study includes soil management while FAOSTAT does not.
[d] This only includes energy from natural gas
[e] This also includes disposal of food packaging

On-farm Production

As demonstrated, the U.S. animal emissions, along with on-farm energy consumption and GHG emissions estimated by FAOSTAT, closely align with the estimates in this study. However, because this analysis includes emissions from soil management under crop emissions, the crop emissions in this study (125.4 MMT CO_{2e}) are significantly higher than those estimated by FAOSTAT (47.8 MMT CO_{2e}). Additionally, the emissions from fertilizer and pesticide manufacturing estimated by this study (9.2 MMT CO_{2e}) differ markedly from those estimated by FAOSTAT (22.9 MMT CO_{2e}), despite FAOSTAT only considering fertilizer manufacturing. This discrepancy arises primarily from the differing methodologies employed.

FAOSTAT first calculated the total fertilizer production in the U.S., applied a global food share coefficient to estimate food-related fertilizer production, and then used product-specific emission factors to estimate the emissions. In contrast, this study began with USDA chemical application data [56], estimated the average chemical application rate for different food commodities to account for crops with missing direct application data. From there the total chemical products applied was calculated, closely matching other U.S. fertilizer use estimations, and then approximated the total energy consumed using energy intensities for different energy sources, and finally applied emission factors to the total energy consumption to estimate GHG emissions.

The authors were able to trace FAOSTAT's source data for total fertilizer production in the United States [94], which closely matches the total fertilizer production data provided by the USDA. However, the FAO utilized food share coefficients to estimate total fertilizer used for food agriculture, compared to total produced, which appear to differ slightly compared to this study. FAOSTAT applied food share coefficients ranging from 0.88 to 0.99 for nitrogen fertilizer applied relative to the total fertilizer manufactured in the United States over the period 1990-2019. When this value was calculated from data used in this study, a coefficient of 0.83 for nitrogen fertilizer was estimated, therefore, FAOSTAT's approach may have resulted in higher estimates of fertilizer application. Additionally, the product-specific energy intensity and emission factors utilized by FAOSTAT were, in general, not provided in the FAOSTAT's methodology notes, therefore the authors are unable to conduct a more in-depth comparison.

Finally, this analysis includes emissions from animal feed manufacturing, which further increases the discrepancy between the on-farm emissions estimated by this study and those reported by FAOSTAT.

Food Manufacturing

Both FAOSTAT and this study estimated energy consumption for food processing and packaging first and then applied emission factors for different energy sources to estimate GHG emissions. The total on-

site energy consumption for this stage estimated by FAO and this study closely matches. However, the emissions from energy consumption differ significantly, with FAOSTAT estimating 94 MMT CO_{2e}, while this study estimates 75.5 MMT CO_{2e}.

The difference between studies is primarily caused by the different energy portfolios assumed by each study. This study utilized the on-site energy mix from the EIA's 2014 and 2018 [75,76], which revealed that 54% of the on-site energy consumed in food manufacturing came from natural gas, 25% from electricity, 7% from coal, 6% from renewable energy, 2% from petroleum, and 5% from other energy sources. In contrast, FAOSTAT's estimation includes 66% from natural gas, 26% from electricity, 5% from coal, and 7% from heat (e.g., purchased steam).

Additionally, FAOSTAT utilized emission factors from the IEA (2013) [95] to estimate GHG emissions from electricity and heat generation and adopted the standard emission factors from the IPCC's 2006 guidelines [96] for other fuel types. Conversely, while this study also used the IPCC's 2006 guidelines [96]for fuels, it adopted EIA's 2016 [26] energy profile for electricity generation data by state (Supplemental Note 2) and applied it to the states in which the energy was used, allowing for regional differences in grid mix to be preserved and tailoring the GHG emissions directly to the year being examined, leading to a lower emission estimation.

Moreover, FAOSTAT estimated the fugitive emissions from food processing to be 8 MMT CO_{2e}, which is much higher than this study's estimation of 2.59 MMT CO_{2e}. The estimation made by this study is applied the ratio of energy used for process cooling of the sector and the total U.S. manufacturing process cooling energy use to the total fugitive emissions from industrial refrigeration in 2016 (9 MMT CO_{2e}) reported by UN Climate Change GHG Inventory Data [74]. However, FAOSTAT does not provide details on the estimation of fugitive emissions from food processing and other activities (e.g., food transport, food retail, and household consumption).

Food Transport/Distribution

Both FAOSTAT and this analysis consider only emissions from domestic food transport. However, unlike FAOSTAT, this analysis estimates the emissions from transportation between the farm gate and manufacturers separately from the distribution of final food products. FAOSTAT's methodology is illustrated by Equation 1, where F and T denote the energy used in food transportation and the total domestic transportation energy consumption in the U.S., respectively, and E represents the total emissions from U.S. domestic transportation. In contrast, this analysis took a bottom-up approach, first estimating food miles between states for truck and rail transport and then applying energy consumption factors and GHG emission factors.

$$Emissions = (F/T)E \quad (1)$$

As shown in the comparison table, FAOSTAT only reported natural gas consumption (2.4 PJ) for food transport and estimated the GHG emissions from energy consumption to be 94 MMT CO_{2e}. However, FAOSTAT's methodology notes that the total food transportation energy consumption in the United States is estimated to be 760 PJ (about 3% of the total U.S. domestic transportation energy consumption), which is comparable to the 641 PJ estimated by this analysis. By applying this factor to the total U.S. transportation emissions in 2018 [97] (1,876.5 MMT CO_{2e}, as reported by the U.S. EIA), the GHG emissions from U.S. food transportation energy use are calculated to be 56.3 MMT CO_{2e}, which is closer to this study's estimation of 46.2 MMT CO_{2e}. Additionally, FAOSTAT estimated the fugitive emissions from food transportation to be 5.3 MMT CO_{2e}, which is much higher than this study's estimation of 1.6 MMT CO_{2e}. However, as shown in Supplementary Note 6, the UN Climate Change GHG Inventory Data [74] reports the total U.S. transportation (including non-food transportation) fugitive emissions to be only 5.89 MMT CO_{2e} in 2016. The authors then estimated fugitive emissions for food and agricultural materials transportation using the ratio of food mass flow (tonnes of food allocated to each state) and the total mass of refrigerated goods transported across the United States in 2016.

W&R and Consumption

The scope of food retail defined by FAOSTAT is equivalent to the W&R plus food services as defined by this study. FAOSTAT estimated energy consumption and GHG emissions at W&R and food services to be 1,429 PJ and 49.8 MMT CO_{2e}, respectively, while this study estimated them to be about 1,000 PJ and 132 MMT CO_{2e}. Similarly, the energy consumption and GHG emissions at household consumption estimated by this study (575 PJ and 65.4 MMT CO_{2e}) and by FAOSTAT (937.5 PJ and 94 MMT CO_{2e}) also differ significantly.

Both FAOSTAT and this study applied emission factors to energy consumption to estimate emissions. However, FAOSTAT used food share factors to determine stage energy consumption (e.g., food-related energy use in commerce and public services represent 6% of the total sector) calculated from EIA 2009 (2012) [98] and/or Canning et al. (2017) [99] and total energy consumption from UNSD energy statistics [100]. In contrast, this study utilized energy consumption data in food retail, food services, and residential cooking, refrigeration, and freezing directly from the EIA's Annual Energy Outlook (2018) [86].

Although the food share for W&R and food services used by FAOSTAT is also derived from EIA data (2012) [98], the food share derived from the data used for this study (EIA AEO 2018 [86]) was estimated to be 11.8%. When comparing data for households, the authors attempted to replicate the FAOSTAT estimations, but values from their methodology did not align with what is currently reported by the UNSD, making comparisons of methodology difficult.

Finally, this study estimated the fugitive emissions from W&R, food services, and households combined to be 28.8 MMT CO2e, differing from FAOSTAT's estimation of 48.1 MMT CO_{2e}. Similar to the other FSC stages, the UN Climate Change GHG Inventory Data reports 45.46 MMT CO_{2e} as the total fugitive emissions from these sectors (i.e., commercial and residential) in the U.S. in 2016. This study estimated the food related fugitive emissions based on this number and the method detailed in Supplemental Note 8.

Waste Management

As shown in Table S 7, FAO estimated significantly lower GHG emissions for food waste management, likely due to the substantially different scope definitions (mentioned above), making a direct comparison challenging.

Overall, both FAOSTAT and this study estimated that U.S. on-farm production (as defined by this study) has the highest GHG emissions among all FSC stages, even though emissions due to soil management are not included by FAOSTAT. The next highest areas are the combination of W&R and consumption stages. Both studies identified food processing as the third largest contributor to GHG emissions from the U.S. FSC, followed by food transport (transportation of raw materials and final food products), and FLW management having the lowest GHG emissions.

Differences in GHG emissions and energy consumption figures between these two studies primarily result from variations in methods and source data used. Additionally, the Emissions Database for Global Atmospheric Research (EDGAR) [9], maintained by the European Commission Joint Research Centre, also estimates GHG emissions from the U.S. agri-food system (using the same definition as FAOSTAT). However, EDGAR does not report energy consumption data, and since this study estimates many emissions figures based on energy consumption, a detailed comparison between this study and EDGAR is challenging. Nonetheless, comparisons between FAOSTAT and EDGAR show general consistency, despite some differences.

Supplementary Notes Reference

1. Rosenzweig, C. *et al.* Climate change responses benefit from a global food system approach. *Nat. Food* **1**, 94–97 (2020).
2. Crippa, M. *et al.* Food Systems are Responsible for a Third of Global Anthropogenic GHG Emissions. *Nat. Food* **2**, 198–209 (2021).
3. Tubiello, F. N. *et al.* Greenhouse gas emissions from food systems: building the evidence base. *Environ. Res. Lett.* **16**, 065007 (2021).
4. Vermeulen, S. J., Campbell, B. M. & Ingram, J. S. I. Climate change and food systems. *Annu. Rev. Environ. Resour.* **37**, 195–222 (2012).
5. Pelletier, N. Life Cycle Thinking, Measurement and Management for Food System Sustainability. *Environ. Sci. Technol.* **49**, 7515–7519 (2015).
6. Loboguerrero, A. M. *et al.* Food and Earth Systems: Priorities for Climate Change Adaptation and Mitigation for Agriculture and Food Systems. *Sustainability* **11**, 1372 (2019).
7. Xu, X. *et al.* Global greenhouse gas emissions from animal-based foods are twice those of plant-based foods.
8. Food and Agriculture Organization of the United Nations. FAOSTAT - Food and agriculture data. https://www.fao.org/faostat/en/#data.
9. Crippa, M., Guizzardi, D., Solazzo, E., Leip, A. & Tubiello, F. N. EDGAR-FOOD_v6. 2265486 Bytes figshare https://doi.org/10.6084/M9.FIGSHARE.17067449 (2021).
10. Steinhart, J. S. & Steinhart, C. E. Energy Use in the U. S. Food System. *Science* **184**, 307–316 (1974).
11. Canning, P., Charles, A., Huang, S. & Polenske, K. R. *Energy Use in the U.S. Food System*. http://www.ers.usda.gov/publications/pub-details/?pubid=46377.
12. Powell, J. T., Townsend, T. G. & Zimmerman, J. B. Estimates of solid waste disposal rates and reduction targets for landfill gas emissions. *Nat. Clim. Change* **6**, 162–165 (2016).

13. Pagani, M., De Menna, F., Johnson, T. G. & Vittuari, M. Impacts and costs of embodied and nutritional energy of food losses in the US food system: farming and processing (Part A). *J. Clean. Prod.* **244**, (2020).
14. Vittuari, M., Pagani, M., Johnson, T. G. & De Menna, F. Impacts and costs of embodied and nutritional energy of food waste in the US food system: Distribution and consumption (Part B). *J. Clean. Prod.* **252**, 119857 (2020).
15. Cuéllar, A. D. & Webber, M. E. Wasted Food, Wasted Energy: The Embedded Energy in Food Waste in the United States. *Environ. Sci. Technol.* **44**, 6464–6469 (2010).
16. Birney, C. I., Franklin, K. F., Davidson, F. T. & Webber, M. E. An assessment of individual foodprints attributed to diets and food waste in the United States. *Environ. Res. Lett.* **12**, 105008 (2017).
17. Bozeman, J. F., Ashton, W. S. & Theis, T. L. Distinguishing Environmental Impacts of Household Food-Spending Patterns Among U.S. Demographic Groups. *Environ. Eng. Sci.* **36**, 763–777 (2019).
18. Canning, P., Rehkamp, S., Hitaj, C. & Peters, C. J. *Resource Requirements of Food Demand in the United States*. http://www.ers.usda.gov/publications/pub-details/?pubid=98400 (2020).
19. Read, Q. D. *et al.* Assessing the environmental impacts of halving food loss and waste along the food supply chain. *Sci. Total Environ.* **712**, 136255 (2020).
20. United States Environmental Protection Agency. *Inventory of U.S. Greenhouse Gas Emissions and Sinks: 1990-2021*. https://www.epa.gov/ghgemissions/inventory-us-greenhouse-gas-emissions-and-sinks-1990-2021 (2023).
21. United States Environmental Protection Agency. From Farm to Kitchen: The Environmental Impacts of U.S. Food Waste. https://www.epa.gov/land-research/farm-kitchen-environmental-impacts-us-food-waste (2021).
22. Heller, M. C. & Keoleian, G. A. Greenhouse Gas Emission Estimates of U.S. Dietary Choices and Food Loss. *J. Ind. Ecol.* **19**, 391–401 (2015).

23. Heller, M. C., Willits-Smith, A., Meyer, R., Keoleian, G. A. & Rose, D. Greenhouse gas emissions and energy use associated with production of individual self-selected US diets. *Environ. Res. Lett.* **13**, 044004 (2018).

24. Guo, X., Broeze, J., Groot, J. J., Axmann, H. & Vollebregt, M. A Worldwide Hotspot Analysis on Food Loss and Waste, Associated Greenhouse Gas Emissions, and Protein Losses. *Sustainability* **12**, 7488 (2020).

25. United States Energy Information Administration. Electricity - Historical State Data. https://www.eia.gov/electricity/data/state/.

26. United States Energy Information Administration. Monthly Energy Review. https://www.eia.gov/totalenergy/data/monthly/pdf/sec12_7.pdf (2022).

27. United States Environmental Protection Agency. GHG Emission Factors Hub. https://www.epa.gov/climateleadership/ghg-emission-factors-hub (2015).

28. United States Energy Information Administration. Annual Energy Outlook 2023. https://www.eia.gov/outlooks/aeo/index.php.

29. United States Department of Agriculture. QuickStats: Fuels, Incl lubricants - Expense by NAICS Classification and State. https://quickstats.nass.usda.gov/results/687F1CA3-1B12-3463-88A6-49C8D2E8170D (2017).

30. United States Department of Agriculture. QuickStats: Fuels, Expense, Measured in $, by Region. (2017).

31. United States Department of Agriculture. ARMS III Farm Production Regions Map. https://www.nass.usda.gov/Charts_and_Maps/Farm_Production_Expenditures/reg_map_c.php.

32. United States Energy Information Administration. Hawaii - State Energy Profile Analysis. https://www.eia.gov/state/analysis.php?sid=HI.

33. United States Energy Information Administration. Alaska - State Energy Profile Analysis. https://www.eia.gov/state/seds/data.php?incfile=/state/seds/sep_use/ind/use_ind_AK.html&sid=AK.

34. United States Energy Information Administration. Wholesale Propane Weekly Heating Oil and Propane Prices (October - March). https://www.eia.gov/dnav/pet/pet_pri_wfr_a_epllpa_pwr_dpgal_w.htm (2017).
35. United States Energy Information Administration. Natural Gas Industrial Price. https://www.eia.gov/dnav/ng/ng_pri_sum_a_epg0_pin_dmcf_a.htm (2017).
36. United States Energy Information Administration. Gasoline and Diesel Fuel Update. https://www.eia.gov/petroleum/gasdiesel/index.php (2017).
37. United States Energy Information Administration. West Coast less California Gasoline and Diesel Retail Prices. https://www.eia.gov/dnav/pet/pet_pri_gnd_dcus_r5xca_w.htm (2017).
38. United States Department of Agriculture. QuickStats: Ag Services, Utilities - Expense, Measured in$, by State and NAICS Classification. (2017).
39. United States Department of Agriculture. QuickStats: Water, Irrigation, Source - Off-farm - Expense, Measured in $, by State. (2017).
40. United States Energy Information Administration. State Electricity Profiles. https://www.eia.gov/electricity/state/archive/2017/ (2017).
41. United States Department of Agriculture. QuickStats: Production, Measured in Tons or Pounds, National and by State. (2017).
42. Dong, W. *et al.* A Framework to Quantify Mass Flow and Assess Food Loss and Waste in The US Food Supply Chain. *Commun. Earth Environ.* **3**, 1–11 (2022).
43. American Feed Industry Assocation. *2016 U.S. Animal Food Consumption Report*. (2017).
44. United States Department of Agriculture. Annual Agruicutural Statistics. 1–9 (2019).
45. United States Department of Agriculture. QuickStats: Sugar/Hay/Peanut - Production. (2017).
46. United States Department of Agriculture. QuickStats: Energy, Renewable - Number of Operations. (2017).
47. United States Energy Information Administration. Annual Energy Outlook 2020. https://www.eia.gov/outlooks/aeo/ (2020).

48. United States Environmental Protection Agency. Inventory of U.S. Greenhouse Gas Emissions and Sinks: 1990-2019. https://www.epa.gov/ghgemissions/inventory-us-greenhouse-gas-emissions-and-sinks-1990-2019 (2021).
49. United States Department of Agriculture. QuickStas: Rice - Acres Planted.
50. United States Department of Agriculture. QuickStas: Ag Land, Cropland - Acres.
51. United States Department of Agriculture. QuickStats: Ag Land, Pastureland - Acres. https://quickstats.nass.usda.gov/results/B6649227-83E2-324B-88DB-A65882E1548E.
52. United States Department of Agriculture. QuickStas: Field Crop Totals, Principal, INCL Potatos - Acres Planted.
53. United States Department of Agriculture. QuickStas: Inventories of livestock. https://quickstats.nass.usda.gov/results/8A59A10A-4F50-39C2-B2EE-D3444AB1B7C3.
54. United States Department of Agriculture. QuickStats: Acres Harvested, by State and Commodity. https://quickstats.nass.usda.gov/results/01FBEC8C-4A2A-3466-900B-618A33BC0052.
55. United States Department of Agriculture. QuickStats: Acres Bearing, by State and Commodity. https://quickstats.nass.usda.gov/results/0C01361A-CD1F-363B-9821-DA1FA90BDA0A.
56. United States Department of Agriculture. QuickStats: Chemical & Fertilizer Applications, by State and Commodity. https://quickstats.nass.usda.gov/results/994EE3D2-8AFC-39C7-9C30-2538354A9596.
57. Thelin, G. P. & Stone, W. W. *USGS Scientific Investigation Report 2013–5009: Estimation of Annual Agricultural Pesticide Use for Counties of the Conterminous United States, 1992–2009*. https://pubs.usgs.gov/sir/2013/5009/ (2013).
58. Ludemann, C. I., Gruere, A., Heffer, P. & Dobermann, A. Global data on fertilizer use by crop and by country. *Sci. Data* **9**, 501 (2022).
59. USDA QuickStats: Sugar/Hay/Peanut - Production. https://quickstats.nass.usda.gov/results/1B8A1202-6376-3887-8CC2-3AA5EA40B0CF.

60. Statista Research Department. Consumption of Agricultural Fertilizers in the United States from 2010 to 2021, by Nutrient (in Million Metric Tons). *Statista* https://www.statista.com/statistics/1330021/fertilizer-consumption-by-nutrient-us/ (2023).
61. Gellings, C. W. Energy Efficiency in Fertilizer Production and Use. 5.
62. West, T. O. & Marland, G. A Synthesis of Carbon Sequestration, Carbon Emissions, and Net Carbon Flux in Agriculture: Comparing Tillage Practices in the United States. *Agric. Ecosyst. Environ.* **91**, 217–232 (2002).
63. Bhat, M. G., English, B. C., Turhollow, A. F. & Nyangito, H. O. *Energy in Synthetic Fertilizers and Pesticides: Revisited. Final Project Report*. https://www.osti.gov/biblio/10120269 (1994) doi:10.2172/10120269.
64. Audsley, E., Stacey, K., DJPaRSONS & Williams, A. *Estimation of the Greenhouse Gas Emissions from Agricultural Pesticide Manufacture and Use*. (2009). doi:10.13140/RG.2.1.5095.3122.
65. United States Environmental Protection Agency. *Pesticides Industry Sales and Usage*. https://www.epa.gov/sites/default/files/2017-01/documents/pesticides-industry-sales-usage-2016_0.pdf (2017).
66. United States Department of Agriculture. *Annual Agricultural Statistics*. (2019).
67. Douglas, E. M. *Boundaries, Areas, Geographic Centers and Altitudes of the United States and the Several States with a Brief Record of Important Changes in Their Territory*. *Bulletin* https://pubs.usgs.gov/publication/b689 (1923) doi:10.3133/b689.
68. Dettling, J., Tu, Q., Faist, M., DelDuce, A. & Mandlebaum, S. *A Comparative Life Cycle Assessment of Plant-Based Foods and Meat Foods*. https://www.morningstarfarms.com/content/dam/NorthAmerica/morningstarfarms/pdf/MSFPlantBasedLCAReport_2016-04-10_Final.pdf (2016).
69. United States Census Bureau. State Population Totals and Components of Change: 2010-2019. *Census.gov* https://www.census.gov/data/tables/time-series/demo/popest/2010s-state-total.html.

70. Andrea Corona, Alexi Ernstoff, Carolina Segato, & Melissa Zgola. *Greenhouse Gas Emissions of Food Waste: Methodology*. chrome-extension://efaidnbmnnnibpcajpcglclefindmkaj/https://refed.org/downloads/quantis-ghg-methodology-vfinal-2020-11-03.pdf (2020).
71. Hwang, H.-L., Lim, H., Chin, S.-M., Wang, C. (Ross) & Wilson, B. *Exploring the Use of FHWA Truck Traffic Volume and Weight Data to Support National Truck Freight Mobility Study*. https://www.osti.gov/biblio/1615795 (2019) doi:10.2172/1615795.
72. Davis, S. C. & Boundy, R. G. *Transportation Energy Data Book: Edition 39*. https://www.osti.gov/biblio/1767864 (2021) doi:10.2172/1767864.
73. Rai, A. Energy demand and environmental impacts of food transport refrigeration and energy reduction methods during temperature-controlled distribution. (Brunel University London, 2019).
74. United Nations Climate Change. Greenhouse Gas Inventory Data - Detailed data by Party. https://di.unfccc.int/detailed_data_by_party.
75. United States Energy Information Administration. 2018 MECS Survey Data. https://www.eia.gov/outlooks/aeo/data/browser/#/?id=43-AEO2018&region=0-0&cases=ref2018&start=2016&end=2018&f=A&linechart=ref2018-d121317a.4-43-AEO2018&map=&sourcekey=0.
76. United States Energy Information Administration. 2014 MECS Survey Data. https://www.eia.gov/consumption/manufacturing/data/2014/.
77. Industrial Assessment Center. IAC Download Data. (2023).
78. Manufacturers' News, Inc. National Manufacturing Industry Database. https://www.mni.net/solutions/ (2020).
79. United States Energy Information Administration. Manufacturing Energy Consumption Survey (MECS) - Components of Onsite Generation of Electricity by Manufacturing Indsutry and Region. https://www.eia.gov/consumption/manufacturing/data/2018/ (2018).

80. United States Energy Information Administration. Manufacturing Energy Consumption Survey (MECS) - Table 4.2: Offsite-Produced Fuel Consumption by Manufacturing Industry and Region. https://www.eia.gov/consumption/manufacturing/data/2018/ (2018).
81. United States Energy Information Administration. Manufacturing Energy Consumption Survey (MECS) - Table 3.2: Fuel Consumption by Manufacturing Industry and Region. https://www.eia.gov/consumption/manufacturing/data/2018/ (2018).
82. United States Energy Information Administration. Manufacturing Energy Consumption Survey (MECS) - Table 5.4: Energy Consumption as a Fuel by End Use by Manufacturing Industry. https://www.eia.gov/consumption/manufacturing/data/2018/ (2018).
83. United States Energy Information Administration. Commercial Buildings Energy Consumption Survey (CBECS) Data. https://www.eia.gov/consumption/commercial/data/2018/.
84. United States Department of Agriculture. QuickStats: Cold Storage Capacity, Warehouse, General, Gross - Capacity, Measured in Cubic ft and Number of Warehouses. https://quickstats.nass.usda.gov/results/0A3BC3A4-DB73-3051-831F-EC8691D2DAA0.
85. CBRE. Food on Demand Series: Cold Storage Logistics Unpacked. https://www.cbre.us/research-and-reports/US-Food-in-Demand-Series-Cold-Storage-Logistics-Unpacked-May-2019.
86. United States Energy Information Administration. Commercial Sector Energy Consumption, Floorspace, and Equipment Efficiency, and Distributed Generation. https://www.eia.gov/consumption/commercial/ (2018).
87. United States Energy Information Administration. Residential Sector Key Indicators and Consumption. https://www.eia.gov/outlooks/aeo/data/browser/#/?id=4-AEO2018&cases=ref2018&sourcekey=0 (2018).
88. United States Department of Agriculture. Food-at-Home Monthly Area Prices. https://www.ers.usda.gov/data-products/food-at-home-monthly-area-prices/.

89. United States Bureau of Economic Analysis (BEA). Personal consumption expenditures (PCE) by major type of product. https://apps.bea.gov/iTable/?reqid=19&step=3&isuri=1&1921=survey&1903=84 (2016).

90. United States Environmental Protection Agency. Documentation for Greenhouse Gas Emission and Energy Factors Used in the Waste Reduction Model (WARM) Organic Materials Chapters. (2016).

91. Tsang, Y. F. *et al.* Production of bioplastic through food waste valorization. *Environ. Int.* **127**, 625–644 (2019).

92. Gironi, F. & Piemonte, V. Bioplastics and petroleum-based plastics: Strengths and weaknesses. *Energy Sources Part Recovery Util. Environ. Eff.* **33**, 1949–1959 (2011).

93. Morris, J., Brown, S., Matthews, H. S. & Cotton, M. Evaluation of Climate, Energy, and Soils Impacts of Selected Food Discards Management Systems. *Or. State Dep. Environ. Qual.* (2014).

94. FAOSTAT. FAOSTAT: Fertilizers by Product. (2021).

95. International Energy Agency. *CO2 Emissions from Fuel Combustion 2013*. (Organisation for Economic Co-operation and Development, Paris, 2013).

96. Eggleston, H. S., Buendia, L., Miwa, K., Ngara, T. & Tanabe, K. 2006 IPCC Guidelines for National Greenhouse Gas Inventories. (2006).

97. US EPA. Fast Facts on Transportation Greenhouse Gas Emissions. https://www.epa.gov/greenvehicles/fast-facts-transportation-greenhouse-gas-emissions (2015).

98. United States Energy Information Administration. Commercial Sector Energy Consumption, 2009. (2012).

99. *The Role of Fossil Fuels in the U.S. Food System and the American Diet*. (2017). doi:10.22004/ag.econ.262187.

100. United Nations. Energy Statistics Database. http://data.un.org/Explorer.aspx?%E2%80%8Cd=EDATA (2021).
